# Biomolecular condensates as stress sensors and modulators of bacterial signaling

**DOI:** 10.1371/journal.ppat.1012413

**Published:** 2024-08-15

**Authors:** Moeka Sasazawa, Dylan T. Tomares, W. Seth Childers, Saumya Saurabh

**Affiliations:** 1 Department of Chemistry, New York University, New York, New York, United States of America; 2 Department of Chemistry, University of Pittsburgh, Pittsburgh, Pennsylvania, United States of America; Carnegie Mellon University, UNITED STATES OF AMERICA

## Abstract

Microbes exhibit remarkable adaptability to environmental fluctuations. Signaling mechanisms, such as two-component systems and secondary messengers, have long been recognized as critical for sensing and responding to environmental cues. However, recent research has illuminated the potential of a physical adaptation mechanism in signaling—phase separation, which may represent a ubiquitous mechanism for compartmentalizing biochemistry within the cytoplasm in the context of bacteria that frequently lack membrane-bound organelles. This review considers the broader prospect that phase separation may play critical roles as rapid stress sensing and response mechanisms within pathogens. It is well established that weak multivalent interactions between disordered regions, coiled-coils, and other structured domains can form condensates via phase separation and be regulated by specific environmental parameters in some cases. The process of phase separation itself acts as a responsive sensor, influenced by changes in protein concentration, posttranslational modifications, temperature, salts, pH, and oxidative stresses. This environmentally triggered phase separation can, in turn, regulate the functions of recruited biomolecules, providing a rapid response to stressful conditions. As examples, we describe biochemical pathways organized by condensates that are essential for cell physiology and exhibit signaling features. These include proteins that organize and modify the chromosome (Dps, Hu, SSB), regulate the decay, and modification of RNA (RNase E, Hfq, Rho, RNA polymerase), those involved in signal transduction (PopZ, PodJ, and SpmX) and stress response (aggresomes and polyphosphate granules). We also summarize the potential of proteins within pathogens to function as condensates and the potential and challenges in targeting biomolecular condensates for next-generation antimicrobial therapeutics. Together, this review illuminates the emerging significance of biomolecular condensates in microbial signaling, stress responses, and regulation of cell physiology and provides a framework for microbiologists to consider the function of biomolecular condensates in microbial adaptation and response to diverse environmental conditions.

## Author summary

Microbes have evolved to adapt rapidly to environmental changes, utilizing complex signaling mechanisms. Among these, formation of biomolecular condensates by phase separation has recently been identified as a potential method for bacteria, typically lacking membrane-bound organelles, to organize their internal biochemistry. Condensates are viscoelastic assemblies responsive to small fluctuations in the environment and function as effective sensors and rapid response systems to environmental stresses. The review highlights the role of phase separation in organizing essential biochemical pathways within cells, including those managing DNA architecture, RNA modification and decay, signal transduction, and stress responses. The discussion also extends to the potential of targeting these protein condensates in pathogens with novel antimicrobial therapies. This emerging understanding positions biomolecular condensates as crucial elements in microbial signaling and adaptation, offering a new perspective for microbiological research into the regulation of cellular responses and pathogen behavior.

## Introduction

**Fig 1:**
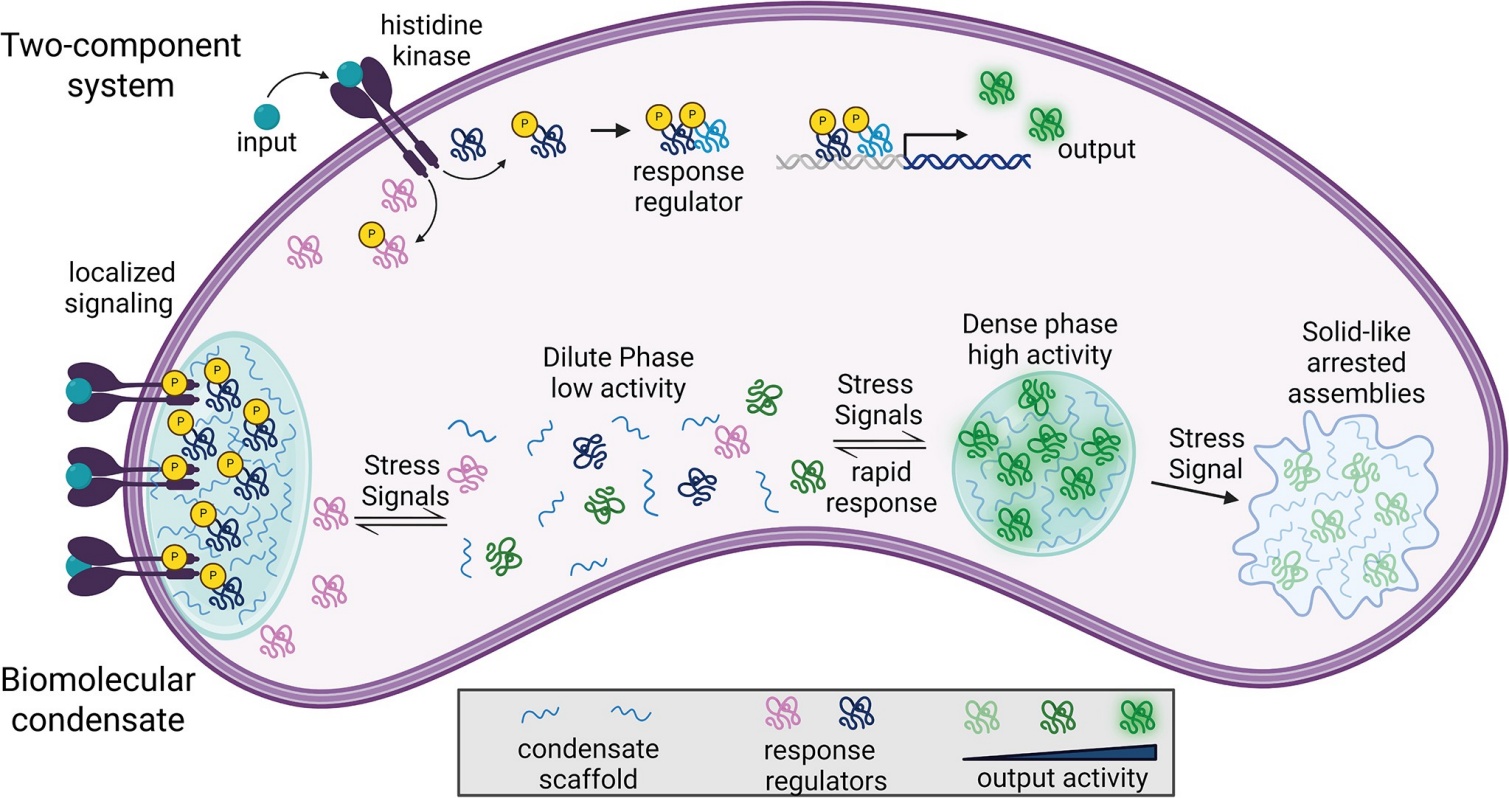
Comparison of signaling via two-component systems vs. biomolecular condensates. TCS (top) represents classical sensing mechanisms in microbes whereby a membrane localized histidine kinase receives an environmental signal and transduces it via response regulators ultimately resulting in regulation of gene expression that occurs over long time scales. Biomolecular condensates (bottom) are an attractive mechanism by which signaling molecules such as kinases can be localized in the presence of environmental fluctuations including stress. The activity and function of localized enzymes within fluid condensates can change rapidly in the presence of stressors. The viscoelastic properties of condensates themselves can be regulated by environmental signals including stress that can transform condensates into solid-like arrested assemblies. Created with BioRender.com.

The success of microbes as symbionts and pathogens relies upon adaptive mechanisms that allow them to sense and adapt to environmental fluctuations over short and evolutionary time scales. Given pressures for survival and adaptation, microbes have evolved a broad range of stress sensing and response mechanisms. The stress response in bacteria is a series of cellular processes that are activated in response to various environmental stressors, such as changes in temperature, pH, osmolarity, nutrient availability, and the presence of toxins. These include sensory inputs from one- or two-component systems [1,2] and signal outputs in the form of changes in second messenger concentrations [3,4]. One-component systems consist of a single protein that directly functions as both the sensor and the regulator, while two-component systems are more complex and consist of 2 proteins: a sensor kinase and a response regulator. The sensor kinase detects the environmental stimulus and autophosphorylates, transferring the phosphate group to the response regulator. The phosphorylated response regulator then modulates gene expression to counteract the stress (Fig 1). Both one- and two-component systems have their limitations. One-component systems are limited in sensitivity and range of detection. Two-component systems provide greater specificity and amplification of signal and access to signals in the periplasmic space. However, two-component systems are more complex and energy-intensive, with potential issues of cross-talk among parallel two-component systems. The issue of crosstalk may be significant without leveraging spatial organization within a cell or a localized complex. Both systems commonly exert their control through gene expression, motility, or proteolysis. Due to inherent limitations on the rate of gene expression, one- and two-component systems exhibit slow regulatory changes compared to fast environmental fluctuations. For fast signaling responses, bacteria exploit second messengers such as guanosine pentaphosphate (pppGpp) and cyclic-di-GMP (cdG) as rapid response mechanisms [5,6]. Another rapid response mechanism was observed in yeast where reversible self-assembly of proteins under heat stress aids the cell’s adaptive response [7,8]. These reversible assemblies form by a process called phase separation that partitions proteins and macromolecules into a dilute and dense phase.

Phase separation can lead to the formation of membraneless organelles called biomolecular condensates, which differ from lipid or protein-bound compartments in many ways [9,10]. Unlike lipid or protein-bound compartments where the boundary is defined by a distinct chemical specie (such as lipid or protein), the condensate boundary is physical in nature, defined by a phase change [11]. A second distinction can be made based on macromolecular transport into and out of organelles. While membrane-bound organelles often rely on active transport systems, enrichment within condensates are then governed by diffusion and capture mechanisms [11–13]. Further, biomolecular condensates exhibit a wide range of viscoelastic properties, dissolution, demixing, and macromolecular exchange in response to the physicochemical environment [10,14–17]. The environmentally sensitive phase transitions provide sensory capabilities that static membrane organelles lack. Finally, condensates often contain proteins and nucleic acids with conformationally flexible regions that utilize weak, multivalent interactions to promote self-assembly and phase separation [18]. By exploiting tunable, multivalent interactions, biomolecular condensate can modulate enzyme activity through mass action, tune substrate specificity through co-localization, or inhibit enzyme functions through the solidification of assemblies (Fig 1) [9]. Similar to rapid responses initiated by secondary messengers, environmentally triggered phase transition provides another avenue for rapid control of biochemical pathways.

Phase separation was initially observed in eukaryotic germ-line RNA processing granules that continuously fuse and disassociate [14]. Notably, when subjected to shear stress, germ-granules in a nucleolus flow, drip, and fuse as liquids. Since this groundbreaking discovery, phase separation has been identified as a prevalent mechanism governing the organization of macromolecules in eukaryotic cells [9]. It plays a crucial role in vital processes such as DNA maintenance and replication (including the nucleolus and DNA damage foci) and RNA processing and decay (such as p-bodies and stress granules) [9]. Spatiotemporal control of signal transduction in a cell is crucial to achieving specificity in biochemical reactions that rely simply on diffusion and reactive collisions. Phase separation has been shown to organize multicomponent networks in response to the activation of signaling proteins in immune cells. Salient examples include the T-cell receptor phosphorylation-dependent phase transition of downstream signaling proteins [19] and the DNA-dependent activation of the cyclic GMP-AMP synthase (cGAS)-stimulator of interferon genes (STING) signaling pathway [20]. It is proposed that phase separation of key signaling proteins enhances their local concentration, thereby increasing the probability of interactions and reducing the noise associated with signaling reactions that mediate adaptive responses [21].

**Fig 2:**
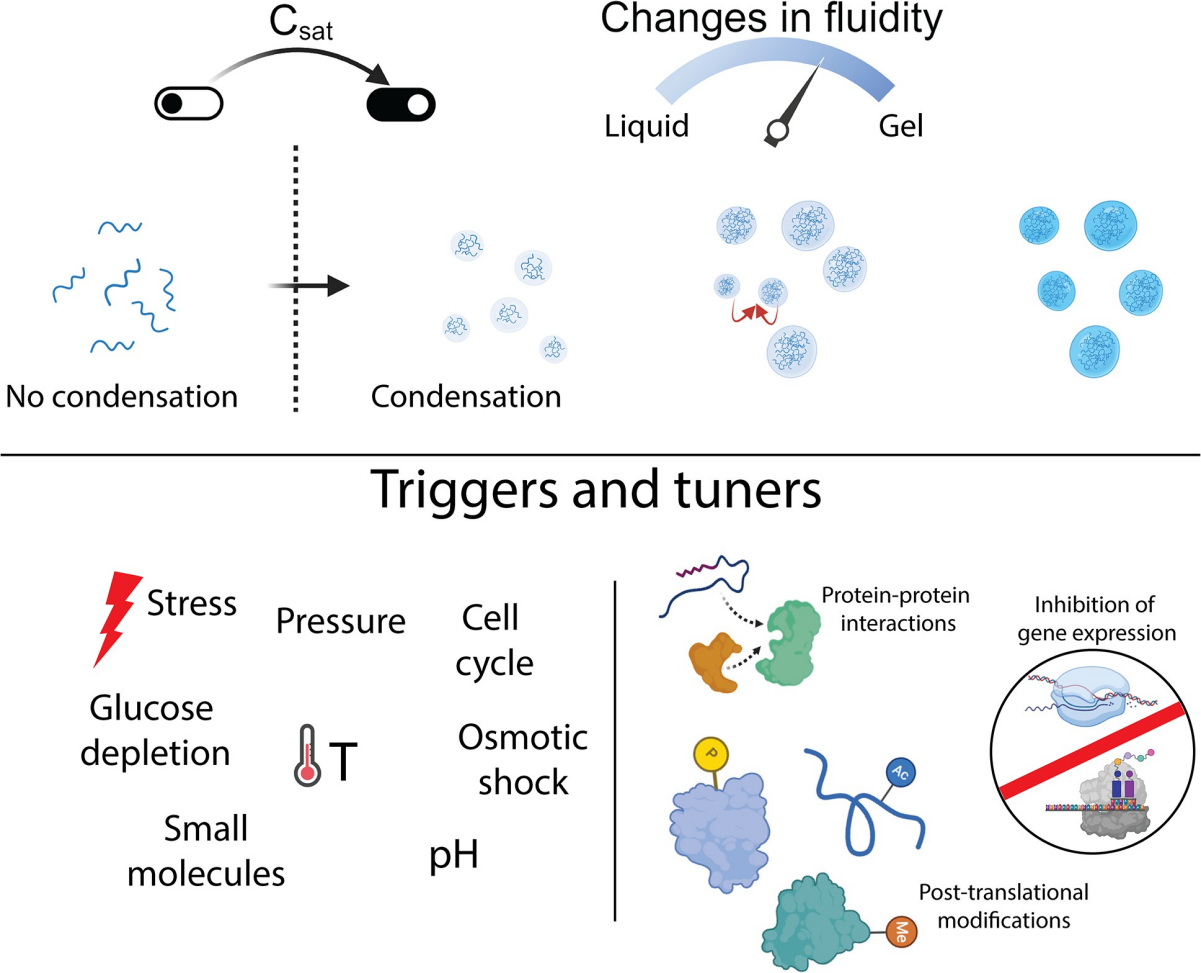
Triggers and tuners of condensates. Formation of condensates is triggered when the local concentration of macromolecules reaches a level called C_sat_. C_sat_ can be tuned by metabolic, thermal, or mechanical stressors. Once formed, condensates can grow by the addition of more condensate-forming macromolecules or by fusion of fluid condensates. Condensate formation and growth is tightly regulated by a variety of external factors such as fluctuations in pressure, temperature, pH, nutrient levels, osmolarity, and small molecule concentrations. On the other hand, intrinsic factors such as protein–protein interactions, disruptions in gene expression, and protein posttranslational modifications are key regulators of condensate properties and functions. Created with BioRender.com.

Since bacteria often lack membrane-bound organelles to separate various biological processes, phase separation may present a ubiquitous mechanism for self-organizing biomolecules in the bacterial cytoplasm. In line with this idea, many bacterial protein assemblies have been proposed to be biomolecular condensates in the last 5 years [16,22–31]. Similar to their eukaryotic counterparts, bacterial condensate-forming proteins are composed of both structured domains and intrinsically disordered regions (IDRs), and environmental parameters control their assembly and disassembly. Owing to physical and chemical rules that govern phase transitions, condensates can act as sensors and responders to their environment [32]. The first of these rules is the presence of a critical concentration (C_sat_) above which condensate formation takes place. In the bacterial cell, condensation of proteins and nucleic acids can be achieved via cell cycle-dependent changes in expression and proteolysis, or environmental fluctuations in pH or temperature, thereby shifting the equilibrium from mostly dilute monomeric macromolecules into a more dense phase. Once formed, condensate growth can occur by the addition of more monomeric macromolecules or fusion events between fluid condensates. Growth mechanisms of condensates are governed by molecular interactions within the condensates that depend on posttranslational modifications, temperature, salts, pH, and oxidative stresses that can act as signals. Further, the viscoelastic properties of the dense phase are often modulated by IDRs [25,29], which has a direct impact on molecular diffusion. Coupled with this, phase separation can result in a change in biomolecular activities and functions in the dilute versus dense phases (Fig 2). It’s in this vein that we consider that phase separation may represent a ubiquitous signaling strategy that can be used in sensing stress or as an adaptive output stress response. This review considers the broad potential of phase separation for microbial signaling and stress response to complement the more well-studied signaling modalities.

### The relevance of condensate formation in bacteria that often lack membrane-bound organelles

**Fig 3:**
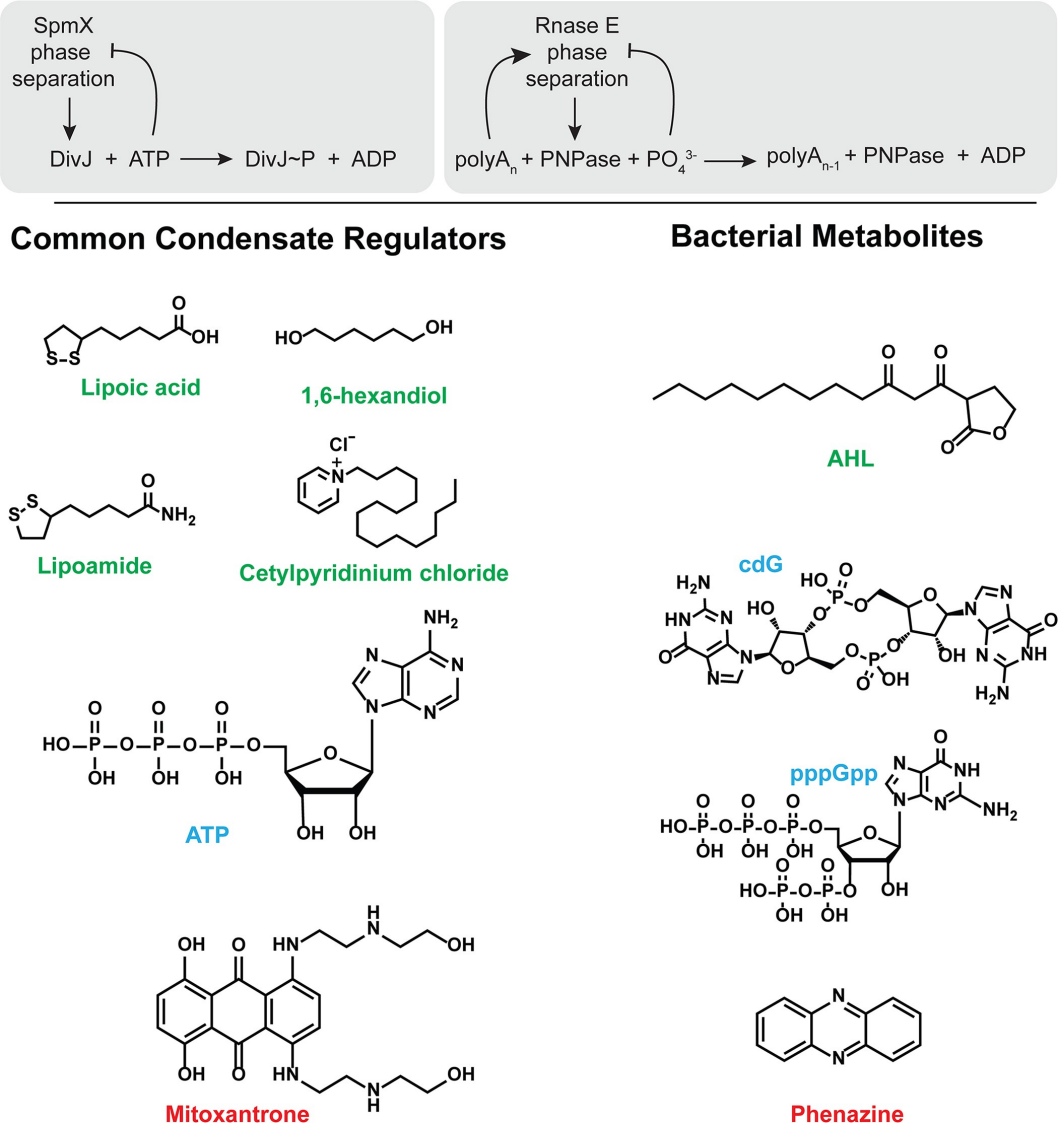
Small molecule-based regulation of condensate properties and function. (Top) Salient examples of condensates from the aquatic bacterium *Caulobacter crescentus* that can be regulated by small molecule metabolites. SpmX is an IDP that forms polar condensates that localize the Histidine kinase DivJ. SpmX condensate formation is promoted under ATP depletion while dissolution is favored under high ATP concentrations. This ATP-dependent feedback is in turn exploited to regulate DivJ kinase activity in response to substrate availability. RNase E condensate regulates PNPase activity but is inhibited by phosphate, which is the substrate of this enzyme. RNase E phase separation is regulated by positive feedback from one substrate (polyA) and negative feedback from the other (phosphate). (Bottom) A repertoire of molecules that can tune condensate formation and disassembly. Lipoic acid, lipoamide, cetylpyridinium chloride, ATP, and hexanediol have been shown to dissolve biomolecular condensates. Other molecules with similar structural features such as antibiotics (Mitoxantrone), second messengers (cyclic di-GMP), and quorum-sensing ligands (AHLs) can be putative modulators of bacterial condensates.

To answer whether phase separation is relevant for bacteria, it is imperative to understand the universal nature of the physicochemical determinants of condensate formation. The intrinsic determinants of condensate formation are multivalent biological polymers such as proteins and nucleic acids. Unlike purely stoichiometric protein complexes composed of high-affinity interactions, condensates rely upon a combination of multivalent structured domain interactions and weak electrostatic, aromatic, or hydrophobic interactions via IDRs [9]. A distinction can be made between the essential proteins that act as “scaffolds” to form the condensate and the “client” or “member” proteins/nucleic acids that partition within the condensate [33]. In addition to the condensate-forming scaffold proteins, interacting proteins can contribute to multivalency and reduce the C_sat_ for phase separation [34,35]. Owing to the ensemble of conformations of their components, condensate assembly and function are sensitive to extrinsic physical and chemical parameters [36]. Key physical parameters that modulate scaffold interactions within condensates are temperature, crowding, and pressure. Among chemical parameters, dielectric constant, pH, ionic strength, and amphiphilic molecules affect the assembly of condensates. Additionally, a repertoire of small molecules including key metabolites has been shown to modulate condensate formation and dissolution (Fig 3). For example, ATP has been shown to be a dissolver for many condensates at physiologically relevant concentrations. This effect is exploited by bacterial condensates to control the activity of a member kinase that regulates cell division [16]. Another example of metabolic regulation is seen with the bacterial ribonucleoprotein-bodies (BR-bodies), where phosphate levels tune condensate formation and activity of enzymes within the condensate. In addition to metabolites, aromatic and hydrophobic molecules such as lipoic acid, cetylpyridinium chloride, and aliphatic polyols have been shown to dissolve condensates. It remains to be seen whether microbe-relevant small molecules such as the ubiquitous second messenger cdG or those involved in quorum sensing such as acyl homoserine lactones (AHLs) can also act as selective modulators of bacterial condensates [37,38].

In the cellular context, protein modifications add a key regulatory layer in condensates as they can change the context of multivalent interactions, thereby modulating client partitioning, scaffold assembly, and dissolution [39]. Together, the combined effect of intrinsic and extrinsic parameters enables condensates to exhibit a spectrum of shapes (spheres, fibers, amorphous solids) [40], viscoelastic properties (fluid, gel, solid) [41], and functions (signal activation, inhibition, buffering) [9]. Both extrinsic and intrinsic regulators of condensate formation are present across all kingdoms of life. Therefore, condensate formation could represent a general mechanism for stress adaptation. This argument has been supported by the many observations of bacterial proteins that form phase-separated, viscoelastic condensates in vitro [16,22–25,27–31,41]. However, the evidence for the mechanisms underlying condensate assembly, modulation of material properties, and their function is still limited for bacteria due to technical limitations in experimental strategies to assess condensate formation in bacteria [42]. In this direction, application of the recently proposed experimental framework to assess the formation and emergent properties of phase-separating proteins in bacteria [43] will be critical for exploiting the sensing and response mechanisms of condensates in microbiology. Nevertheless, the repertoire of proposed bacterial condensates grows, with several examples from host–bacterial systems that could be promising avenues for antimicrobial therapeutics, provided a connection between condensate formation, function, and cell fitness can be established [33,44]. We summarize the condensates reviewed in Table 1, highlighting the stress signal they respond to, and the primary evidence for phase separation. In the next section, we highlight selected systems in microbes where connections between condensate formation and their function in vivo are alluded to.

**Table 1:** Condensates, their function and evidence for phase separation.

	Stressor/signal sensed	Response modulation	Experimental evidence
Dps	Nutrient starvation (stationary phase) [28]	DNA compaction while allowing for transcription [28]	In vitro reconstitution of condensates exhibiting viscoelasticity and demixing [28,48]
Hu	Oxidative and pH damage [49]	Modulates nucleoid compaction [49]	In vitro reconstitution of condensates—viscoelasticity and demixing [48]
SSB	DNA damage [55]	DNA repair [55]	In vitro reconstitution, dissolution, viscoelasticity [56]
BR-body	Stresses leading to increased RNA in the cell, ethanol, heat shock [22], phosphate [65]	Regulation of RNA degradation [22]	Fusion of clusters, RNA-dependent cluster size observed via super-resolution microscopy [22,58]
Rho	Nutrient starvation [66]	Transcription [66]	Cryo-ET with domain mutants, in vitro reconstitution [66]
PopZ	Nutrient starvation, cell cycle-dependent localization [13,16,72]	Chromosome segregation, phosphosignaling [26,72]	Cryo-ET observation of membraneless boundary, demixing, fusion, single-molecule studies of diffusion [13,26,72,78]
SpmX	ATP depletion [16]	Kinase activity modulation [16,80]	Cryo-ET and live cell observation of demixing. Single-molecule studies of diffusion with domain mutants [16,78]
PodJ	Cell cycle-dependent expression and proteolysis [88,89]	Periodic phosphatase localization and activity modulation as a cell-cycle checkpoint [31,90]	Client localization by phase separating domains in vivo, Interfacial demixing in vitro [31,90]
ABC transporter	Cell cycle-dependent phosphorylation [24]	Phosphorylation-dependent assembly of the transporter [24]	Fusion of foci in a heterologous expression system [24]
Aggresome	ATP depletion [94]	Recovery from dormancy [94]	Fusion of foci in live cells [95]
Polyphosphate granules	Nitrogen starvation, MgCl_2_ fluctuation [101,105,114]	Cell cycle exit under starvation [114]	Cryo-EM, transmission electron microscopy [114,125]

Evidence considered: Observation of membraneless boundary, fusion of clusters in vivo, demixing, measurements of diffusion or viscoelasticity on domain mutants, phase separation response to relevant fluctuations in vivo and in vitro.

## Phase separation as an adaptive response for nucleoid protection during stress

The nucleoid, which is dynamic, organized, and compact, changes as cells grow, divide, and become dormant. The nucleoid must be accessible for DNA replication, chromosome segregation, and gene transcription. While the nucleoid itself was observed to form a phase-separated system within the bacterial cytoplasm [45], recent studies have provided some evidence that nucleoid-associated proteins and DNA repair pathways can form biomolecular condensates to promote genome organization across evolution [46]. This phase separation organizes large stretches of DNA into a condensate state. This state of nucleoprotein material provides stress protection from nucleases, reactive oxygen species, and UV damage. Notably, the weak interactions within these liquid-like materials may enable the diffusion of transcriptional machinery and access to DNA-binding sites, facilitating gene regulation. In contrast, more solid-like compaction strategies may impede transcription. Together, the spectrum of material properties exhibited by nucleoprotein condensates offers the potential for fast adaptive responses to nutrient deprivation, oxidative stress, and posttranslational modifications, influencing nucleoid properties. This review focuses on 3 nucleoid-associated condensates (Dps, Hu) and the DNA repair complexes (SSB).

### Dps condensates compact chromosomes while allowing transcription machinery access

Under nutritional stress posed by the stationary phase, the *Escherichia coli* genome undergoes compaction by expression of Dps (DNA protection during starvation). Studies by Meyer and colleagues made the earliest assertion of a bacteria forming biomolecular condensates [28]. The foundation of this model was observations from in vitro single-molecule magnetic tweezers that showed a condensed phase of DNA upon Dps binding [28]. A previous study by the same authors demonstrated in vitro that DNA compaction was persistent over long time scales despite the dynamic binding and unbinding of Dps protein. However, phase separation was not invoked as a mechanism then [47]. Recent in vitro studies have also observed liquid-like Dps-DNA condensates [48]. However, whether or how the material properties of Dps-DNA assemblies impact their function in vivo remains unanswered. A hint for this function comes from Meyer and colleagues, who demonstrated that transcription is not blocked despite chromosome compaction by Dps [28]. The study demonstrates that phase separation may provide an elegant solution of high DNA compaction coupled with unperturbed access for transcription, which would be essential for microbes to exit starvation conditions when more favorable growth persists [28].

### Hu condensates protect the chromosome from stress and respond to phage-produced proteins

A second major nucleoid-associated protein is the Hu (heat unstable) protein, which binds nonspecifically to DNA, albeit with some preference for ds-DNA break repair sites and AT-rich regions. In *Helicobacter pylori*, Hu protects the nucleoid from oxidative and pH damage [49]. In vivo, super-resolution imaging of Hu-eYFP in *C*. *crescentus* and *E*. *coli* observed largely diffuse signals with sub-diffraction clusters of approximately 150 nm appearing in pre-divisional cells [50,51]. Past experiments have shown that Hu impact upon compaction may be highly responsive to posttranslational modification interaction with other proteins or small molecules. For example, Hu-DNA interactions in *Mycobacterium tuberculosis* is regulated by a posttranslational lysine acetylation that decreases DNA binding and genome compaction [52]. Bacteriophage proteins may also regulate the functions of Hu; for example, the phage protein Gp46 forms a complex with Hu and prevents DNA binding [53]. Recent in vitro studies have shown that Hu forms condensates with DNA and exhibit demixing [48]. Future studies, preferably following Moerner and colleagues’ high-resolution approaches [50,54], will be needed to demonstrate how posttranslational modifications and phage-associated proteins impact Hu phase separation in vivo and in vitro. Such studies could provide evidence that genome compaction mediated by phase separation is an adaptation to stress exposure.

### SSB condensates coordinate the functions of DNA repair

The integrity of the chromosomal DNA of a bacterium in the host’s harsh environment depends on robust repair mechanisms. Single-stranded DNA binding proteins (SSBs) bind to exposed single-stranded DNA (ssDNA) regions, preventing them from reannealing while mitigating ssDNA degradation and damage. Super-resolution imaging of *E*. *coli* via structured illumination microscopy showed that SSB localized as foci associated with the inner membrane while biochemical assays revealed low micromolar affinity for phosphatidylglycerol. Upon induction of DNA damage induced by mitomycin C, SSB localizes as foci within the nucleoid [55]. In vitro studies demonstrated SSB phase separation at concentrations similar to endogenous levels, where the ratio of SSB to ssDNA regulates the formation and dissolution of SSB condensates [56]. From these studies, the authors propose that SSB serves as a scaffold for DNA repair enzymes that are subsequently deployed when exposed to high levels of ssDNA substrates. Among outstanding questions is how phase separation affects the partitioning of SSB from the lipid-rich membrane to a DNA-bound state in vivo. Studies of DNA repair condensates across domains of life have shown varying material properties [57]. Understanding the relationships between in vivo material properties of DNA repair condensates and the functions of DNA repair will be a critical area of future research and could represent an untapped antimicrobial strategy.

## The RNA degradosome is a biomolecular condensate

In eukaryotic cell biology, biomolecular condensates are involved in diverse RNA biochemical processes, including processing, splicing, degradation, and ribosome biogenesis. Biomolecular condensates provide strategies to store and release RNAs in response to changing environmental conditions controlling critical gene expression programs. In bacteria, biomolecular condensates have been implicated in RNA decay and transcription termination.

The endoribonuclease RNase E had several features suggesting it could be a bacterial biomolecular condensate. First, *C*. *crescentus* RNase E functions as a degradosome scaffold by recruiting the exoribonucleases PNPase and RNase D, the helicase RhlB, and the TCA cycle metabolic enzyme aconitase thereby mimicking some of the same functions as eukaryotic p-bodies and stress granules [22,58]. Second, RNase E is composed of a long C-terminal IDR enriched in acidic and basic residues [59]. Interestingly, while the primary sequence is non-conserved, a polyampholyte-charged block patterning was observed between different RNase E orthologs across bacterial species. This was reminiscent of multi-block copolymer designs in polymer chemistry [60]. Finally, past cell biology studies have shown that RNase E forms foci in cells [61]. These features provided the foundation to consider RNase E in a bacterial biomolecular condensate framework.

Early experimental support for this idea came from live cell single-molecule tracking experiments where RNase E molecules exhibited slow and fast diffusing populations. Confined RNase E molecules diffused almost 2 orders of magnitude slower than the mobile population [62]. A second key piece of evidence came from time-lapse imaging of RNase E- YFP imaging at endogenous expression levels that showed rapid fusion of short-lived foci in living cells. The presence of RNA substrates stimulates the formation of RNase E condensates. At the same time, depletion of RNAs from cells by treatment with the RNA polymerase inhibitor rifampicin led to a decrease in RNase E cluster size, suggestive of reversibility [62]. A third key in vitro study showed that RNase E’s C-terminal IDR phase separated as biomolecular condensates dependent upon protein concentration and salt levels, supporting the observation that foci formation in the cell is dependent upon the C-terminal IDR.

In addition, Hfq assemblies, termed H-bodies, can also form independent of RNase E in the absence of RNAs. Single-molecule tracking revealed confined diffusion of Hfq monomers within these assemblies [63]. Condensation of HfQ within the cytoplasm and at the cell poles is crucial for their function as RNA chaperones under stress induced by high osmolarity or stationary phase [64]. These findings indicate that the composition of ribonucleoproteins can be highly variable, which may imply diverse cell functions, particularly under stress conditions.

### BR-bodies co-localize multi-step RNA decay and prevent the build-up of toxic RNA intermediates

Differential centrifugation can also separate biomolecular condensates from other proteins in a lysate. Using this strategy, it was demonstrated that BR-bodies were selectively permeable to long and unstructured RNAs and excluded structured RNAs such as tRNAs and rRNAs [58]. Within BR-bodies, the degradosome enzymes work together in a two-step process to break down RNAs. Many RNAs are largely unstructured but can contain some RNA secondary hairpins that limit the exoribonuclease activity of PNPase. So, in many cases, RNase E makes the first rate-limiting cut, which generates RNA intermediates that can serve as better substrates for the downstream PNPase enzyme. Strains lacking RNase E’s C-terminal IDR showed an increase in observable RNA decay intermediates that are typically not observable in strains with full-length RNase E. This suggests that one of the primary functions of RNase E is the colocalization of RNase E, PNPase, and RNA intermediates to limit the half-life of RNA decay intermediates and prevent any toxicity they may introduce. A comparison of 4 transcripts by qRT-PCR revealed that RNase E variants that could phase separate had shorter RNA half-lives in the 0.51 to 0.81 min range than the N-terminal domain that could not phase separate with half-lives of 1.5 to 2.7 min [58]. In vitro studies show that phase separation also accelerates the activity of PNPase about 3.4-fold compared to the PNPase in the dilute state [65]. This suggests the phase-separated environment accelerates RNA decay in vivo and in vitro. More broadly, BR-bodies highlight an example where biomolecular condensates can play critical roles in multi-enzyme cascades to tightly control the half-life of intermediates to avoid toxicity. Preliminary studies also suggest BR-bodies may be responsive to multiple stresses. For example, in 5% ethanol stress BR-body number and intensity increase. Moreover, RNase E’s C-terminal domain, that is critical for phase separation, is also critical for high fitness when cells are exposed to 5% ethanol stress [58,65]. These observations provide initial support for BR-bodies in ethanol stress response. Therefore, an exciting research avenue will be to study how these stresses modulate the material properties and functions of BR-bodies.

### An IDR within Rho is required for colonization by a gut microbe under nutrient scarcity

Nutrient-dependent phase separation is emerging as a common theme. Another possible area of RNA regulation by phase separation is transcriptional termination regulated by Rho. Colonization of the murine gut by the *B*. *thetaiotaomicron* (Bt) depends upon a ~300 amino acid N-terminal IDR of Rho, which is required for phase separation in vitro and cluster formation in vivo. This IDR is unique in BtRho and absent in other commonly studied Rho homologs. In vitro studies demonstrated an IDR and RNA-dependent phase separation [66]. Low RNA to Rho ratios stimulate Rho phase separation in vitro, whereas high RNA to Rho ratios lead to the dissolution of Rho condensates. While these results present interesting hypotheses about the role of Rho phase separation in *B*. *thetaiotaomicron*, the in vivo evidence must be considered critically. For example, fewer clusters were observed via diffraction-limited microscopy of immunostained *B*. *thetaiotaomicron* expressing Rho without its IDR compared to full-length, WT Rho, which is interpreted as a proxy for phase separation. An increase in the number of clusters is observed when the cells are starved of glucose, and treatment with the compound 1,6-hexanediol decreases the number of observed clusters independent of glucose levels. Cryo-electron tomography (Cryo-ET) data is also presented to support the idea that WT Rho forms clusters in bacteria harvested from the mice gut, unlike the mutant lacking the IDR, where no clusters were observed.

Given that immunofluorescence shows a relative decrease in clusters in the same strains, alternative interpretations of the cryo-ET data must be considered. Further, use of compounds such as 1,6-hexanediol could affect the membrane permeability and DNA organization, thereby necessitating the reconsideration of these results carefully [16,67,68]. Despite limited evidence for in vivo phase separation, this study demonstrates the importance of the IDR for gene expression and fitness during nutrient starvation. Given that the Rho-IDR is not found in pathogens such as *E*. *coli*, it is unclear whether bacteria universally utilize this domain during host colonization. Similarly, the *C*. *crescentus* Rho contains only a short 19 residue IDR. A *C*. *crescentus* BR-body client proteomic study identified Rho as a BR-body client, and observed Rho was incapable of forming a biomolecular condensates independent of RNase E [69,70]. Future studies will be needed to solidify the in vivo evidence for phase separation and discern its unique context-dependent advantages for Rho for the mammalian gut microbiome versus other biochemical strategies used for transcription termination.

## Condensates and signaling proteins are logically wired as a stress response mechanism

Biomolecular condensates are also intertwined with the process of signal transduction. We will discuss examples of PopZ, PodJ, and SpmX proteins that show condensate regulation of signaling enzyme localization and activity. Uniquely, the sensitivity of condensate material properties to stresses acts as an upstream sensory element for two-component systems that commonly sit at the start of signaling cascades. In comparison, studies of *Mycobacterium tuberculosis* (Mtb) ABC transporter Rv1747 demonstrate phosphorylation-dependent regulation of condensate formation. These examples suggest condensates can be involved as sensors or internal components within signaling cascades.

### A polar signaling hub modulates client diffusion

Polar localization of proteins by diffusion and capture is a hallmark of bacterial cells. In many cases, the captured proteins can localize to the poles via curvature sensing or nucleoid exclusion [13,71–73]. One of the best-studied cases of polar localization due to phase separation is the pole-organizing protein (PopZ) in *Caulobacter crescentus* [13,72]. Asymmetric cell division of the alphaproteobacteria *C*. *crescentus* and its cattle pathogen homolog *Brucella abortus* rely on 2 compositionally distinct polar condensates that PopZ organizes [74,75]. As the name implies, PopZ is crucial in scaffolding over a dozen cell cycle-associated clients at 2 distinct cell poles [75]. One of its earliest functions identified was to tether the nucleoid-binding protein ParB to the cell poles after chromosome segregation [13,72,76,77]. PopZ’s polar foci formation mechanism was shown to occur by nucleoid occlusion well before the emergence of the biomolecular condensate field [72]. The earliest cryo-ET images of overexpressed PopZ show that the PopZ condensates form a distinct zone with a membraneless boundary that excludes ribosomes [72,77]. Evidence for PopZ phase separation is further strengthened by observations of fusion and viscoelastic properties when expressed heterologously in yeast and mammalian cells [25,72].

While the earliest evidence of PopZ exhibiting a confined polar population was observed by single protein tracking, more recent super-resolution microscopy experiments showed PopZ to form a distinct cell pole localized zone [13,16,26,76]. These observations have been substantiated via recent development in correlative electron tomography and single-molecule microscopy at cryogenic temperatures [78]. Modest fluorescent recovery after photobleaching (FRAP) demonstrated that PopZ assemblies were more fluid than the solid-like IcsA assemblies but less fluid than its client protein CckA and diffuse GFP [75]. Single-molecule tracking experiments showed that PopZ was significantly immobilized at the cell pole, and phosphosignaling client protein diffusion slowed within the PopZ condensate [13,26]. Interestingly, non-client proteins such as eYFP and fPIF-eYFP diffused through the cytoplasm but were excluded from the PopZ condensates [26]. Experiments exploiting in vitro reconstitution and heterologous expression in mammalian cells further support the idea that PopZ alone is a scaffold and can form liquid-like droplets [16,25]. Thus, ample evidence exists that PopZ forms a membraneless organelle that regulates signaling pathways and chromosome tethering.

Compositional diversification of these 2 condensates is achieved by 2 accessory scaffolds: PodJ and SpmX [79,80]. At the new cell pole, PodJ organizes a chemical environment at the new cell pole that activates CckA kinase activity. A second scaffold, SpmX, resides at the old cell pole and facilitates a chemical environment that represses CckA signaling activity. Here, we consider what is known about the phase properties of PodJ and SpmX and how they exert control over the signaling chemistry in each condensate.

### SpmX provides ATP-dependent kinase signaling to ward off nutritional stress

During differentiation of *C*. *crescentus* from a planktonic to a sessile state, a membrane localized intrinsically disordered protein (IDP) called SpmX localizes to the PopZ condensate [80]. SpmX directly interacts with the histidine kinase DivJ that is required for the division of the differentiated cell [80,81]. SpmX has been shown to form oligomeric assemblies that require coordination between its lysozyme homology domain and its IDR [82]. In vitro reconstitution and super-resolution microscopy support the idea that SpmX forms a biomolecular condensate at the cell pole [16]. To explore the relationship between SpmX and PopZ condensates, correlative cryo-ET with single-molecule imaging was applied to reveal that SpmX and PopZ formed demixed condensates at the cell pole, a seminal observation that was also verified via in vitro reconstitution and live cell super-resolution microscopy [16,78].

SpmX is an evolutionarily conserved developmental factor in alphaproteobacteria and its function has been tied to the positioning of the stalk, a polar appendage crucial for nutrient acquisition [83,84]. While these were the first studies highlighting the hypervariability of a bacterial IDR during evolution, the function this unstructured domain remained unclear. SpmX-IDR was shown to interact with the DivJ kinase in vitro [82]. Interestingly, SpmX condensates exhibited a response to physiological levels of ATP, which has been suggested as a functional link to modulation of DivJ kinase activity. ATP is a substrate for kinases and has been shown to solubilize stress-responsive condensates in eukaryotes by a mechanism that is not fully understood [85,86]. Low ATP levels promote SpmX condensation, increasing DivJ’s local concentration and kinase activity while high levels of ATP dissolve SpmX condensates [16]. In line with these experiments, the SpmX-IDR is dispensable in nutrient-rich conditions but critical for DivJ regulation under nutrient-poor conditions in live cells [16]. ATP’s role in promoting condensate dissolution is likely a general mechanism for the delocalization of enzymes that are clients of condensates, as is evident from studies of circadian cycle-connected protein assemblies in *Cyanobacteria* [87].

### PodJ condensates provide an intermediate cell-cycle checkpoint for spatial control of signaling

PodJ provides an example of a condensate that is wired as an intermediate part in the cell-cycle pathway of *C*. *crescentus*. As opposed to responding to external stress signals, PodJ is expressed and proteolyzed in each cell cycle [79,88,89]. In this manner, PodJ biomolecular condensate formation and dissolution act as a key checkpoint. PodJ has been shown to accumulate as a foci in *C*. *crescentus* strains that lack the PopZ scaffold, indicating that PodJ can form cell pole-associated foci without the assistance of PopZ. In vivo FRAP studies of overexpressed PodJ have shown rapid recovery of PodJ within 30 s. Notably, 50% of the population is immobile, which is a proxy for the degree of protein localization within the condensate [31]. Further demonstration of PodJ’s condensate properties in vivo has been limited by its interaction with the PopZ scaffold, which also supports its polar localization. In vitro studies of PodJ have shown that the N-terminal region composed of an IDR and a coiled-coil oligomerization domain also forms liquid-like condensates [31,90]. PodJ recruits one of its major clients, the bifunctional histidine kinase PleC, through interactions with its PAS sensory domain. In vitro, studies indicated that this PodJ-PleC interaction leads to robust repression of PleC kinase activity. Thus, PodJ condenses and mediates the localization of PleC and simultaneously represses PleC’s signaling functions. This coupling of allostery and recruitment provides a strategy for spatial regulation of enzyme function.

Current examples of condensate regulation by two-component systems have been linked to asymmetric cell division and cell polarity. The PodJ and SpmX studies highlight conceptual links between phase separation and the stimulation of histidine kinase signaling. These studies highlight the potential that two-component systems may leverage the phase separation process to sense an array of cellular stresses. Moreover, it raises critical questions about how phase separation regulates other steps of bacterial signaling. For example, do condensate environments impact phosphotransfer specificity through the selective recruitment of select response regulators and exclusion of other response regulators? Moreover, it’s unknown if other major types of bacterial signaling are impacted by phase separation. For example, it’s been speculated that localized c-di-GMP is prevalent in bacteria, and biomolecular condensate may provide a mechanism to regulate localized pools of c-di-GMP or other secondary messengers [91,92].

### Phosphosignaling regulates ABC transporter condensates in *Mycobacterium tuberculosis*

The *Mycobacterium turberculosis* (Mtb) ABC transporter Rv1747 interacts with the universal stress protein Rv2623 and is significant for Mtb’s growth and virulence in hosts [93]. Rv1747 is one of the best-studied systems within a pathogen where phase separation is tuned by posttranslational modifications [24]. The cytoplasmic regulatory module of Rv1747 which consists of 2 phosphothreonine-binding domains connected by an IDR. It was shown that upon phosphorylation, this domain can form liquid-like condensates that are reversible and responsive to environmental changes. Both phosphorylation and dephosphorylation processes regulate the phase separation of Rv1747, mediated by a specific serine/threonine kinase and a phosphatase in Mtb. This regulation affects the clustering and activity of the transporter within the mycobacterial membrane. Experimentation in live cells and supported lipid bilayers revealed clustering of the Rv1747 regulatory module, suggesting that self-assembly of this protein also occurs in more complex and biologically relevant environments. It’s been proposed that phase separation mediated clustering of the ABC transporter could increase transport efficiency due to allosteric activity regulation, amplify signals by selectively filtering substrates, and form scaffolded complexes with cell wall biosynthesis pathways. These results can be further strengthened by future experiments and analyses of Rv1747 clustering profiles and co-localization analyses of the interacting kinases and phosphatases. Regardless, this work implicates phase separation as a fundamental mechanism for the spatial and temporal regulation of protein function in a pathogen, particularly in response to environmental stresses. Future studies could use the lack of clustering as a selection criterion for ABC transporter inhibitors and this mechanism could potentially be targeted for developing new antimicrobial strategies.

## Formation of aggresomes and polyphosphate granule condensates as an output stress response

When exposed to various stresses, bacterial cells leverage biomolecular condensates as survival strategies. Nutrient deprivation stimulates the formation of polyphosphate granules, whereas exposure to antibiotic or heat stresses can trigger the formation of aggresomes. Here, we consider these structures in the framework of biomolecular condensates.

### Aggresome formation during stationary phase as a reversible stress response strategy

A significant strategy utilized by cells to avoid antibiotic stress is to go into a dormant growth state. The degree of dormancy depends upon the formation of protein aggregates that, by white light microscopy, appear as dark foci, termed aggresomes. These aggresomes accumulate insoluble proteins including HslU, Kbl, and AcnB under extended stationary phase, heat shock, and antibiotic treatment. Similar to other bacterial condensates, ATP depletion from cells accelerates the formation of aggresomes and exit from the dormant state requires the aggresome’s dissolution, mediated by the heat shock ATPases DnaK and ClpB [16,87,94]. A follow-up study explored the possibility of phase separation as a mechanism behind formation of aggresomes [95]. In vivo, single-molecule tracking of HslU-EGFP observed diffusion constants (*D*_g_) that range from 0.19 to 0.32 μm^2^/s, exhibiting slower diffusion in stationary phase. Timelapse experiments also observed the fusion of aggresomes over 4 min, suggesting that the assembly may have gel-like properties, although the lack of other diffusion regimes was not explored which makes it challenging to assess the meaning behind these material states [95]. Proteomic studies have identified many of the insoluble proteins in cells, and challenges remain if proteins play clear roles as clients and scaffolds within the aggresomes. Studies in the alphaproteobacteria also show stress-induced aggregates, consistent with aggresomes, that are dissolvable by the functions of DnaK and ClpB [96]. In yeast, work from Drummond and colleagues has demonstrated that acute heat stresses lead to the formation of poly(A)-binding protein (Pab1) condensates, which require ATP-dependent chaperones for dispersal [7,32,97]. Moving forward, the biophysical mechanisms underpinning aggresome phase separation and whether ATP can directly dissolve aggresome formation will be crucial to exploit their effects on dormancy depth for antimicrobial strategies [98].

### Polyphosphate granules as survival strategy under nutritional stress

Polyphosphate (PolyP) is a widely conserved, energy-rich, anionic polymer critical for bacterial virulence and host responses such as blood clotting and amyloidogenesis [99,100]. Its wide conservation can be attributed to its stability across various temperatures, pH, and redox conditions [101]. In the bacterial cell, polyphosphate exists as a polymer of tens to hundreds of phosphate residues linked by “high-energy” phosphoanhydride bonds. Polyphosphate granules were first identified in 1888 in *Saccharomyces cerevisiae* cells [102]. PolyP is visible as a spherical structure reminiscent of condensed droplets in the bacterial cytoplasm, even in the early days of electron microscopy [103,104]. PolyP serves as a storage granule in bacteria that facilitates survival under nutritional stress [101,105,106]. The primary enzyme responsible for the synthesis of PolyP in bacteria is polyphosphate kinase, which catalyzes the transfer of the terminal phosphate of ATP to the growing polyphosphate chain [107,108]. PolyP utilization is carried out by the exopolyphosphatase enzyme that binds to the end of a polyP chain and cleaves the bond between the last 2 phosphate groups in the chain, releasing 1 phosphate molecule at a time [109,110].

Unlike protein-based condensates, polyP granules potentially function as a system where the polymer product acts as the scaffold. This concept is supported by the reconstitution of PolyP using cations and positively charged peptides, which leads to the formation of heterotypic coacervates [111]. PolyP is potentially involved in DNA condensation in *Pseudomonas aeruginosa* and the cyanobacteria *Synechococcus elongatus* [101,112]. Additionally, PolyP has been shown to exhibit bipolar localization in *Corynebacterum glutamicum*, which is enhanced under high concentrations of MgCl_2_, but not due to changes in PolyP regulating gene expression levels [113]. The strongest evidence that PolyP is a condensate comes from live cell fluorescence and electron microscopy observations of the biogenesis as a function of the cell cycle in *P*. *aeruginosa* [114]. PolyP granules reduce in their abundance but grow in their size during the cell cycle, likely by fusion. During these stages, the granules maintain a spherical shape. The observation of spherical structures combined with the metabolic response of PolyP strengthens the claim that this ancient structure is a condensate in vivo [113,114]. PolyP has also been shown to form a three-component condensate with AT-rich DNA and the RNA chaperone protein HfQ in vitro [115]. This observation supports previous electron microscopy data from Murata and colleagues, and has been suggested to contribute to bacterial heterochromatin formation aided by polyP and HfQ. However, chromatin-bound or phase-separated Hfq has not been observed in vivo even using super-resolution microscopy. Future studies should focus on the role of polyP phase separation in virulence and its co-condensation with DNA or RNA-binding proteins. Another interesting question that remains to be answered is whether PolyP can mediate the partitioning of proteins like HfQ between DNA and RNA-dependent roles.

## The untapped potential of intrinsically disordered proteins in microbes

**Fig 4:**
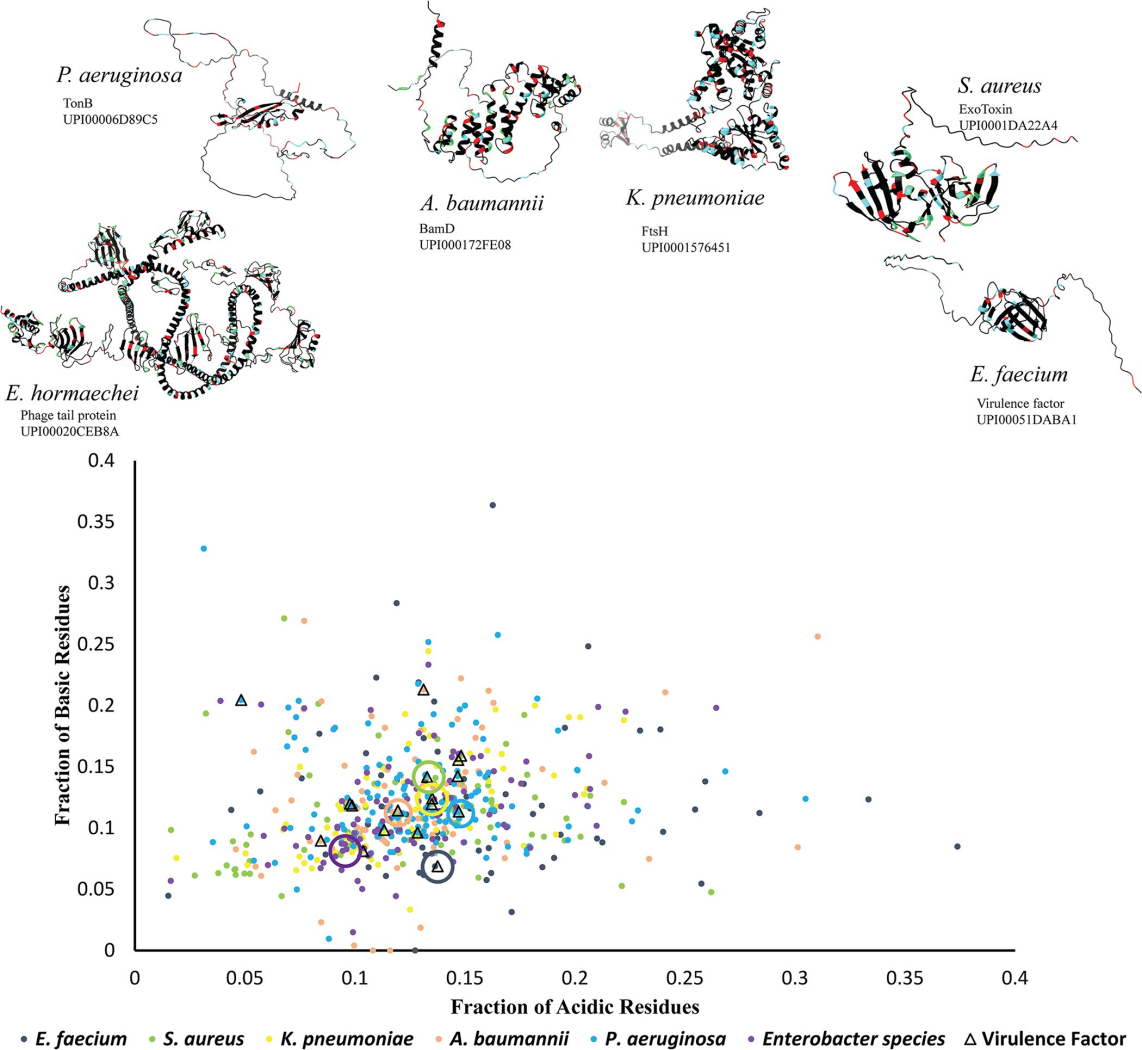
The prevalence of intrinsically disordered proteins in pathogens. Salient examples of virulence factors in ESKAPE pathogens that have significant intrinsically disordered regions are shown. Distribution of IDPs from ESKAPE pathogens, color coded by the species, are shown as a function of the fraction of acidic (x-axis) and basic (y-axis) residues. See supplementary tables and files for more information on the proteins included in the graph.

Studies over the last several years have highlighted over a dozen proteins as condensates in bacteria, raising the question of their breadth and importance for pathogens. Condensates are viscoelastic structures, where interactions between multivalent structured domains control their assembly while IDRs control condensate fluidity [25,29]. Using the presence of IDRs as a proxy for viscoelasticity tuning modules prevalent within condensates, we analyzed IDPs in the proteomes of the ESKAPE pathogens to infer the potential of condensates as antibiotic targets (Figs 4 and S1 and Table 1). For all but 1 pathogen, *P*. *aeruginosa*, we identified approximately 70 to 100 IDPs across proteomes spanning approximately 2,600 to 5,000 proteins. Based on our search criteria, the *Pseudomonas aeruginosa* proteome of over 5,800 has 144 IDPs. Compared to the total number of different types of proteins in these organisms, IDPs represent about 3.5% of the proteome. In comparison, about 23% of proteins within the single-cell eukaryote *S*. *cerevesiae* contain IDRs longer than 50 amino acids. This is consistent with past suggestions that the substantial increase in the abundance of IDPs in a proteome could be correlated with organismal complexity [116–118].

Given that IDPs can self-organize under environmental fluctuations, it could be argued that the more fluctuations an organism experiences in their evolutionary environment, the more utility IDP-mediated pathways could provide. In line with this idea, we observe that pathogens that must adapt to diverse external and host environments have proteomes composed of about 2% to 3% IDRs longer than 50 amino acids. For obligate intracellular bacteria capable of switching between hosts, we observe that despite a dramatic reduction in proteome size, the proportion of IDPs is not significantly reduced. In endosymbionts such as Wolbachia, we observed a dramatic reduction in proteome size yet, approximately 2% of the proteins constituting IDRs longer than 50 residues. This indicates that structured proteins are lost at approximately the same rate as unstructured proteins. Based on these observations, we posit that IDPs may have a crucial role in opportunistic pathogens that need to adapt to a variety of host environments. However, given the conservation of IDRs within bacteria with more minimal streamlined genomes, this suggests bacterial IDRs may play fundamental roles in how cells grow and divide, and likely, a substantial subset could be involved in phase separation.

A framework for considering IDR sequences is in the light of evolution. While evolutionary analysis can be routinely applied to structured proteins, it is difficult to apply them to IDRs because of a lack of sequence and secondary structure conservation. In this direction, new methods have been developed to analyze sequence patterns and their variability across evolution. These analyses set the stage for evo-devo–based studies that include IDPs in bacteria and can also be extended to the evolution of an IDP virulent factor under selection pressures applied by the host. As a first step in this direction, IDR sequences can be categorized into classes based on the fractions of positively charged (f+) and negatively charged (f-) residues, in addition to net charge per residue [119,120]. Our analyses show that most disordered sequences from ESKAPE pathogens occupy the region of weak poly-ionic species and could exhibit a high degree of context-dependent biophysical characteristics. This could have important consequences in the search for compounds to target condensate formation. For example, metabolite mimics that exhibit separated charges could be used to selectively dissolve ionic condensates while not affecting bona fide metabolite-binding proteins.

## Outlook

Biomolecular condensates are now known regulators of many essential processes within bacteria, including pathogens. For example, Hu, RNase E, and ParB are 3 examples of condensate-forming proteins considered antibiotic targets. These assemblies present fundamentally new antibiotic target strategies with significant challenges that typical drug receptor targets do not face. Small molecules could function by dissolving condensates that organize and regulate biochemical pathways. This mode presents substantial challenges as small molecules must disrupt several multivalent protein–protein and protein–nucleic acids “sticker” interactions. Dissolution of condensates by ATP and hexanediol provides a viewpoint of broad-spectrum condensate dissolving approaches, but these mechanisms require significant improvements in potency and specificity. Disruption of phase separation may be approachable if single protein–protein or protein–nucleic interactions regulate the degree of multivalency. For example, given the importance of Hu functions for cell viability and survival, some progress has been made in developing small molecule inhibitors and potential antibiotic properties. Structure-based inhibitors that disrupt the Hu-DNA interaction have been shown to disrupt chromosome compaction in *M*. *tuberculosis*, highlighting Hu as a possible antibiotic target [121]. Secondly, small molecules could function as inhibitors that rewire the composition of condensates by disrupting an essential client protein’s accumulation within condensates. This requires the design of small molecules or peptides that compete with client-scaffold protein–protein interactions. Finally, a third broad mechanism is molecules that function as molecular glues, which harden and severely limit diffusion within condensates. That would involve the design of small molecules that mediate new sticker interactions or new scaffold–client interactions that alter the material properties of condensates. For example, there has been a success in designing small molecules that harden virus-induced inclusion body condensates that serve as sites of virus replication [122]. In this direction, much remains to be gained by utilizing machine learning-based approaches that have been applied in eukaryotic systems and in antibiotic discovery for targeting specific bacterial condensates [123,124]. This would open up avenues of targeting pathogens by physicochemical disruption of condensates, an area that remains promising for fighting the pervading problem of antimicrobial resistance.

By acting as sensors, biomolecular condensates contribute to cellular signaling, gene expression regulation, and stress response pathways. Biomolecular condensates can serve as the most upstream components of signaling pathways by sensing variations in environmental factors, such as temperature, nutrients, pH, or internal metabolites. Their ability to rapidly form, dissolve, and selectively concentrate specific pathways allows cells to fine-tune their responses quickly and cooperatively to internal and external cues. For example, ATP may be a general disaggregase for condensates highlighted by ATP’s dissolution effect on SpmX condensates. Biomolecular condensates can also serve as intermediates in signaling pathways detecting intracellular signaling events through response to posttranslational modifications that stimulate or dissolve condensates. For example, ABC transporter condensates are regulated by phosphorylation, and PodJ condensates are regulated by expression and proteolysis. Finally, biomolecular condensates can mediate output responses by forming phosphate granules that allow cells to adapt to low nutrients or initiate dormant states in response to antibiotic stress with aggresomes. This sensing potential of condensates warrants their cautious investigation to understand how microbes have fully leveraged the phase separation phenomena.

## Supporting information

S1 Fig Bar plots showing Gene Ontology (GO) term analyses of IDPs from ESKAPE pathogens. The numbers above each bar represent the total number of IDPs that were included in the analyses. Each IDP’s GO term annotation was obtained from Uniprot. GO term categories that occupied less than 2% in all species are excluded from the graph.
(PDF)

S1 Table Abundance of IDPs in pathogens, free-living and endosymbiotic microbes. (PDF)

S1 Files Proteome files, codes, and spreadsheets utilized for analyses and making S1 Fig and S1 Table are included as a compressed file with the publisher. They can also be downloaded from a Github repository (https://github.com/saurabhLabNYU/BacterialIDPAnalysis).
(ZIP)

## References

[1] Laub MT. The Role of Two-Component Signal Transduction Systems in Bacterial Stress Responses. Bacterial Stress Responses. John Wiley & Sons, Ltd; 2010. p. 45–58. doi: 10.1128/9781555816841.ch4

[2] Skerker JM, Perchuk BS, Siryaporn A, Lubin EA, Ashenberg O, Goulian M, et al. Rewiring the specificity of two-component signal transduction systems. Cell. 2008;133:1043–1054. doi: 10.1016/j.cell.2008.04.040 18555780 PMC2453690

[3] Pesavento C, Hengge R. Bacterial nucleotide-based second messengers. Curr Opin Microbiol. 2009;12:170–176. doi: 10.1016/j.mib.2009.01.007 19318291

[4] Hengge R, Pruteanu M, Stülke J, Tschowri N, Turgay K. Recent advances and perspectives in nucleotide second messenger signaling in bacteria. microLife. 2023;4:uqad015. doi: 10.1093/femsml/uqad015 37223732 PMC10118264

[5] Boutte CC, Crosson S. The complex logic of stringent response regulation in Caulobacter crescentus: starvation signalling in an oligotrophic environment. Mol Microbiol. 2011;80:695–714. doi: 10.1111/j.1365-2958.2011.07602.x 21338423 PMC3093662

[6] Jenal U, Malone J. Mechanisms of cyclic-di-GMP signaling in bacteria. Annu Rev Genet. 2006;40:385–407. doi: 10.1146/annurev.genet.40.110405.090423 16895465

[7] Wallace EWJ, Kear-Scott JL, Pilipenko EV, Schwartz MH, Laskowski PR, Rojek AE, et al. Reversible, Specific, Active Aggregates of Endogenous Proteins Assemble upon Heat Stress. Cell. 2015;162:1286–1298. doi: 10.1016/j.cell.2015.08.041 26359986 PMC4567705

[8] Keyport Kik S, Christopher D, Glauninger H, Hickernell CW, Bard JAM, Lin KM, et al. An adaptive biomolecular condensation response is conserved across environmentally divergent species. Nat Commun. 2024;15:3127. doi: 10.1038/s41467-024-47355-9 38605014 PMC11009240

[9] Lyon AS, Peeples WB, Rosen MK. A framework for understanding the functions of biomolecular condensates across scales. Nat Rev Mol Cell Biol. 2021;22:215–235. doi: 10.1038/s41580-020-00303-z 33169001 PMC8574987

[10] Alberti S, Hyman AA. Biomolecular condensates at the nexus of cellular stress, protein aggregation disease and ageing. Nat Rev Mol Cell Biol. 2021;22:196–213. doi: 10.1038/s41580-020-00326-6 33510441

[11] Greening C, Lithgow T. Formation and function of bacterial organelles. Nat Rev Microbiol. 2020;18:677–689. doi: 10.1038/s41579-020-0413-0 32710089

[12] Rudner DZ, Pan Q, Losick RM. Evidence that subcellular localization of a bacterial membrane protein is achieved by diffusion and capture. Proc Natl Acad Sci U S A. 2002;99:8701–8706. doi: 10.1073/pnas.132235899 12060714 PMC124362

[13] Bowman GR, Comolli LR, Zhu J, Eckart M, Koenig M, Downing KH, et al. A polymeric protein anchors the chromosomal origin/ParB complex at a bacterial cell pole. Cell. 2008;134:945–955. doi: 10.1016/j.cell.2008.07.015 18805088 PMC2745220

[14] Brangwynne CP, Eckmann CR, Courson DS, Rybarska A, Hoege C, Gharakhani J, et al. Germline P granules are liquid droplets that localize by controlled dissolution/condensation. Science. 2009;324:1729–1732. doi: 10.1126/science.1172046 19460965

[15] Fisher RS, Elbaum-Garfinkle S. Tunable multiphase dynamics of arginine and lysine liquid condensates. Nat Commun. 2020;11:4628. doi: 10.1038/s41467-020-18224-y 32934220 PMC7492283

[16] Saurabh S, Chong TN, Bayas C, Dahlberg PD, Cartwright HN, Moerner WE, et al. ATP-responsive biomolecular condensates tune bacterial kinase signaling. Sci Adv. 2022;8:eabm6570. doi: 10.1126/sciadv.abm6570 35171683 PMC8849385

[17] Feric M, Vaidya N, Harmon TS, Mitrea DM, Zhu L, Richardson TM, et al. Coexisting Liquid Phases Underlie Nucleolar Subcompartments. Cell. 2016;165:1686–1697. doi: 10.1016/j.cell.2016.04.047 27212236 PMC5127388

[18] Choi J-M, Holehouse AS, Pappu RV. Physical Principles Underlying the Complex Biology of Intracellular Phase Transitions. Annu Rev Biophys. 2020;49:107–133. doi: 10.1146/annurev-biophys-121219-081629 32004090 PMC10715172

[19] Su X, Ditlev JA, Hui E, Xing W, Banjade S, Okrut J, et al. Phase separation of signaling molecules promotes T cell receptor signal transduction. Science. 2016;352:595–599. doi: 10.1126/science.aad9964 27056844 PMC4892427

[20] Xiao Q, McAtee CK, Su X. Phase separation in immune signalling. Nat Rev Immunol. 2022;22:188–199. doi: 10.1038/s41577-021-00572-5 34230650 PMC9674404

[21] Klosin A, Oltsch F, Harmon T, Honigmann A, Jülicher F, Hyman AA, et al. Phase separation provides a mechanism to reduce noise in cells. Science. 2020;367:464–468. doi: 10.1126/science.aav6691 31974256

[22] Al-Husini N, Tomares DT, Bitar O, Childers WS, Schrader JM. α-Proteobacterial RNA degradosomes assemble liquid-liquid phase-separated RNP bodies. Mol Cell. 2018;71:1027–1039. e14. 30197298 10.1016/j.molcel.2018.08.003 PMC6151146

[23] Ladouceur A-M, Parmar BS, Biedzinski S, Wall J, Tope SG, Cohn D, et al. Clusters of bacterial RNA polymerase are biomolecular condensates that assemble through liquid–liquid phase separation. Proc Natl Acad Sci U S A. 2020;117:18540–18549. doi: 10.1073/pnas.2005019117 32675239 PMC7414142

[24] Heinkel F, Abraham L, Ko M, Chao J, Bach H, Hui LT, et al. Phase separation and clustering of an ABC transporter in Mycobacterium tuberculosis. Proc Natl Acad Sci U S A. 2019;116:16326–16331. doi: 10.1073/pnas.1820683116 31366629 PMC6697873

[25] Lasker K, Boeynaems S, Lam V, Scholl D, Stainton E, Briner A, et al. The material properties of a bacterial-derived biomolecular condensate tune biological function in natural and synthetic systems. Nat Commun. 2022;13:5643. doi: 10.1038/s41467-022-33221-z 36163138 PMC9512792

[26] Lasker K, von Diezmann L, Zhou X, Ahrens DG, Mann TH, Moerner WE, et al. Selective sequestration of signalling proteins in a membraneless organelle reinforces the spatial regulation of asymmetry in Caulobacter crescentus. Nat Microbiol. 2020;5:418–429. doi: 10.1038/s41564-019-0647-7 31959967 PMC7549192

[27] Ramm B, Schumacher D, Harms A, Heermann T, Klos P, Müller F, et al. Biomolecular condensate drives polymerization and bundling of the bacterial tubulin FtsZ to regulate cell division. Nat Commun. 2023;14:3825. doi: 10.1038/s41467-023-39513-2 37380708 PMC10307791

[28] Janissen R, Arens MM, Vtyurina NN, Rivai Z, Sunday ND, Eslami-Mossallam B, et al. Global DNA compaction in stationary-phase bacteria does not affect transcription. Cell. 2018;174:1188–1199. e14. doi: 10.1016/j.cell.2018.06.049 30057118 PMC6108918

[29] Basalla JL, Mak CA, Byrne JA, Ghalmi M, Hoang Y, Vecchiarelli AG. Dissecting the phase separation and oligomerization activities of the carboxysome positioning protein McdB. Pappu RV, Ron D, Childers WS, editors. Elife. 2023;12:e81362. doi: 10.7554/eLife.81362 37668016 PMC10554743

[30] Babl L, Merino-Salomón A, Kanwa N, Schwille P. Membrane mediated phase separation of the bacterial nucleoid occlusion protein Noc. Sci Rep. 2022;12:17949. doi: 10.1038/s41598-022-22680-5 36289351 PMC9606368

[31] Tan W, Cheng S, Li Y, Li X-Y, Lu N, Sun J, et al. Phase separation modulates the assembly and dynamics of a polarity-related scaffold-signaling hub. Nat Commun. 2022;13:7181. doi: 10.1038/s41467-022-35000-2 36418326 PMC9684454

[32] Yoo H, Triandafillou C, Drummond DA. Cellular sensing by phase separation: Using the process, not just the products. J Biol Chem. 2019;294:7151–7159. doi: 10.1074/jbc.TM118.001191 30877200 PMC6509497

[33] Azaldegui CA, Vecchiarelli AG, Biteen JS. The emergence of phase separation as an organizing principle in bacteria. Biophys J. 2021;120:1123–1138. doi: 10.1016/j.bpj.2020.09.023 33186556 PMC8059088

[34] Li P, Banjade S, Cheng H-C, Kim S, Chen B, Guo L, et al. Phase transitions in the assembly of multivalent signalling proteins. Nature. 2012;483:336–340. doi: 10.1038/nature10879 22398450 PMC3343696

[35] Banjade S, Rosen MK. Phase transitions of multivalent proteins can promote clustering of membrane receptors. Elife. 2014;3:e04123. doi: 10.7554/eLife.04123 25321392 PMC4238058

[36] Moses D, Ginell GM, Holehouse AS, Sukenik S. Intrinsically disordered regions are poised to act as sensors of cellular chemistry. Trends Biochem Sci. 2023;48:1019–1034. doi: 10.1016/j.tibs.2023.08.001 37657994 PMC10840941

[37] Jenal U. Signal transduction mechanisms in Caulobacter crescentus development and cell cycle control. FEMS Microbiol Rev. 2000;24:177–191. doi: 10.1016/S0168-6445(99)00035-2 10717313

[38] Taga ME, Bassler BL. Chemical communication among bacteria. Proc Natl Acad Sci U S A. 2003;100:14549–14554. doi: 10.1073/pnas.1934514100 12949263 PMC304117

[39] Bah A, Forman-Kay JD. Modulation of Intrinsically Disordered Protein Function by Post-translational Modifications. J Biol Chem. 2016;291:6696–6705. doi: 10.1074/jbc.R115.695056 26851279 PMC4807257

[40] Boeynaems S, Holehouse AS, Weinhardt V, Kovacs D, Van Lindt J, Larabell C, et al. Spontaneous driving forces give rise to protein−RNA condensates with coexisting phases and complex material properties. Proc Natl Acad Sci U S A. 2019;116:7889–7898. doi: 10.1073/pnas.1821038116 30926670 PMC6475405

[41] Alshareedah I, Moosa MM, Pham M, Potoyan DA, Banerjee PR. Programmable viscoelasticity in protein-RNA condensates with disordered sticker-spacer polypeptides. Nat Commun. 2021;12:6620. doi: 10.1038/s41467-021-26733-7 34785657 PMC8595643

[42] McSwiggen DT, Mir M, Darzacq X, Tjian R. Evaluating phase separation in live cells: diagnosis, caveats, and functional consequences. Genes Dev. 2019;33:1619–1634. doi: 10.1101/gad.331520.119 31594803 PMC6942051

[43] Hoang Y, Azaldegui CA, Dow RE, Ghalmi M, Biteen JS, Vecchiarelli AG. An experimental framework to assess biomolecular condensates in bacteria. Nat Commun. 2024;15:3222. doi: 10.1038/s41467-024-47330-4 38622124 PMC11018776

[44] Cohan MC, Pappu RV. Making the case for disordered proteins and biomolecular condensates in bacteria. Trends Biochem Sci. 2020;45:668–680. doi: 10.1016/j.tibs.2020.04.011 32456986

[45] Valkenburg JA, Woldringh CL. Phase separation between nucleoid and cytoplasm in Escherichia coli as defined by immersive refractometry. J Bacteriol. 1984;160:1151–1157. doi: 10.1128/jb.160.3.1151-1157.1984 6389508 PMC215833

[46] Feric M, Misteli T. Phase separation in genome organization across evolution. Trends Cell Biol. 2021;31:671–685. doi: 10.1016/j.tcb.2021.03.001 33771451 PMC8286288

[47] Vtyurina NN, Dulin D, Docter MW, Meyer AS, Dekker NH, Abbondanzieri EA. Hysteresis in DNA compaction by Dps is described by an Ising model. Proc Natl Acad Sci U S A. 2016;113:4982–4987. doi: 10.1073/pnas.1521241113 27091987 PMC4983820

[48] Gupta A, Joshi A, Arora K, Mukhopadhyay S, Guptasarma P. The bacterial nucleoid-associated proteins, HU and Dps, condense DNA into context-dependent biphasic or multiphasic complex coacervates. J Biol Chem. 2023:299. doi: 10.1016/j.jbc.2023.104637 36963493 PMC10141540

[49] Wang G, Lo LF, Maier RJ. A histone-like protein of Helicobacter pylori protects DNA from stress damage and aids host colonization. DNA Repair. 2012;11:733–740. doi: 10.1016/j.dnarep.2012.06.006 22776439 PMC3976563

[50] Lee SF, Thompson MA, Schwartz MA, Shapiro L, Moerner W. Super-resolution imaging of the nucleoid-associated protein HU in Caulobacter crescentus. Biophys J. 2011;100:L31–L33. doi: 10.1016/j.bpj.2011.02.022 21463569 PMC3072666

[51] Wang W, Li G-W, Chen C, Xie XS, Zhuang X. Chromosome Organization by a Nucleoid-Associated Protein in Live Bacteria. Science. 2011;333:1445–1449. doi: 10.1126/science.1204697 21903814 PMC3329943

[52] Ghosh S, Padmanabhan B, Anand C, Nagaraja V. Lysine acetylation of the Mycobacterium tuberculosis HU protein modulates its DNA binding and genome organization. Mol Microbiol. 2016;100:577–588. doi: 10.1111/mmi.13339 26817737

[53] Zhang P, Zhao X, Wang Y, Du K, Wang Z, Yu J, et al. Bacteriophage protein Gp46 is a cross-species inhibitor of nucleoid-associated HU proteins. Proc Natl Acad Sci U S A. 2022;119:e2116278119. doi: 10.1073/pnas.2116278119 35193978 PMC8892312

[54] Gahlmann A, Moerner WE. Exploring bacterial cell biology with single-molecule tracking and super-resolution imaging. Nat Rev Microbiol. 2014;12:9–22. doi: 10.1038/nrmicro3154 24336182 PMC3934628

[55] Zhao T, Liu Y, Wang Z, He R, Xiang Zhang J, Xu F, et al. Super-resolution imaging reveals changes in Escherichia coli SSB localization in response to DNA damage. Genes Cells. 2019;24:814–826. doi: 10.1111/gtc.12729 31638317 PMC7065570

[56] Harami GM, Kovács ZJ, Pancsa R, Pálinkás J, Baráth V, Tárnok K, et al. Phase separation by ssDNA binding protein controlled via protein−protein and protein−DNA interactions. Proc Natl Acad Sci U S A. 2020;117:26206–26217. doi: 10.1073/pnas.2000761117 33020264 PMC7584906

[57] Miné-Hattab J, Liu S, Taddei A. Repair foci as liquid phase separation: evidence and limitations. Genes. 2022;13:1846. doi: 10.3390/genes13101846 36292731 PMC9602295

[58] Al-Husini N, Tomares DT, Pfaffenberger ZJ, Muthunayake NS, Samad MA, Zuo T, et al. BR-bodies provide selectively permeable condensates that stimulate mRNA decay and prevent release of decay intermediates. Mol Cell. 2020;78:670–682. e8. doi: 10.1016/j.molcel.2020.04.001 32343944 PMC7245546

[59] Cohan MC, Shinn MK, Lalmansingh JM, Pappu RV. Uncovering Non-random Binary Patterns Within Sequences of Intrinsically Disordered Proteins. J Mol Biol. 2022;434:167373. doi: 10.1016/j.jmb.2021.167373 34863777 PMC10178624

[60] Self JL, Zervoudakis AJ, Peng X, Lenart WR, Macosko CW, Ellison CJ. Linear, Graft, and Beyond: Multiblock Copolymers as Next-Generation Compatibilizers. JACS Au. 2022;2:310–321. doi: 10.1021/jacsau.1c00500 35252981 PMC8889609

[61] Montero Llopis P, Jackson AF, Sliusarenko O, Surovtsev I, Heinritz J, Emonet T, et al. Spatial organization of the flow of genetic information in bacteria. Nature. 2010;466:77–81. doi: 10.1038/nature09152 20562858 PMC2896451

[62] Bayas CA, Wang J, Lee MK, Schrader JM, Shapiro L, Moerner WE. Spatial organization and dynamics of RNase E and ribosomes in Caulobacter crescentus. Proc Natl Acad Sci U S A. 2018;115:E3712–E3721. doi: 10.1073/pnas.1721648115 29610352 PMC5910860

[63] McQuail J, Carpousis AJ, Wigneshweraraj S. The association between Hfq and RNase E in long-term nitrogen-starved Escherichia coli. Mol Microbiol. 2022;117:54–66. doi: 10.1111/mmi.14782 34219284

[64] Goldberger O, Szoke T, Nussbaum-Shochat A, Amster-Choder O. Heterotypic phase separation of Hfq is linked to its roles as an RNA chaperone. Cell Rep. 2022;41:111881. doi: 10.1016/j.celrep.2022.111881 36577380

[65] Collins MJ, Tomares DT, Nandana V, Schrader JM, Childers WS. RNase E biomolecular condensates stimulate PNPase activity. Sci Rep. 2023;13:12937. doi: 10.1038/s41598-023-39565-w 37558691 PMC10412687

[66] Krypotou E, Townsend GE, Gao X, Tachiyama S, Liu J, Pokorzynski ND, et al. Bacteria require phase separation for fitness in the mammalian gut. Science. 2023;379:1149–1156. doi: 10.1126/science.abn7229 36927025 PMC10148683

[67] Itoh Y, Iida S, Tamura S, Nagashima R, Shiraki K, Goto T, et al. 1,6-hexanediol rapidly immobilizes and condenses chromatin in living human cells. Life Sci Alliance. 2021;4:e202001005. doi: 10.26508/lsa.202001005 33536240 PMC7898662

[68] Perdikari TM, Murthy AC, Fawzi NL. Molecular insights into the effect of alkanediols on FUS liquid-liquid phase separation. bioRxiv. 2022:p. 2022.05.05.490812. doi: 10.1101/2022.05.05.490812

[69] Nandana V, Rathnayaka-Mudiyanselage IW, Muthunayake NS, Hatami A, Mousseau CB, Ortiz-Rodríguez LA, et al. The BR-body proteome contains a complex network of protein-protein and protein-RNA interactions. Cell Rep. 2023;42:113229. doi: 10.1016/j.celrep.2023.113229 37815915 PMC10842194

[70] Rathnayaka-Mudiyanselage I, Nandana V, Schrader J. Proteomic composition of eukaryotic and bacterial RNA decay condensates suggests convergent evolution. Curr Opin Microbiol. 2024;79:102467. doi: 10.1016/j.mib.2024.102467 38569418 PMC11162941

[71] Treuner-Lange A, Søgaard-Andersen L. Regulation of cell polarity in bacteria. J Cell Biol. 2014;206:7–17. doi: 10.1083/jcb.201403136 25002676 PMC4085708

[72] Ebersbach G, Briegel A, Jensen GJ, Jacobs-Wagner C. A self-associating protein critical for chromosome attachment, division, and polar organization in caulobacter. Cell. 2008;134:956–968. doi: 10.1016/j.cell.2008.07.016 18805089 PMC2614312

[73] Ramamurthi KS, Losick R. Negative membrane curvature as a cue for subcellular localization of a bacterial protein. Proc Natl Acad Sci U S A. 2009;106:13541–13545. doi: 10.1073/pnas.0906851106 19666580 PMC2726380

[74] Bolle XD, Crosson S, Matroule J-Y, Letesson J-J. Brucella abortus Cell Cycle and Infection Are Coordinated. Trends Microbiol. 2015;23:812–821. doi: 10.1016/j.tim.2015.09.007 26497941 PMC8800490

[75] Holmes JA, Follett SE, Wang H, Meadows CP, Varga K, Bowman GR. Caulobacter PopZ forms an intrinsically disordered hub in organizing bacterial cell poles. Proc Natl Acad Sci U S A. 2016;113:12490–12495. doi: 10.1073/pnas.1602380113 27791060 PMC5098656

[76] Ptacin JL, Gahlmann A, Bowman GR, Perez AM, von Diezmann L, Eckart MR, et al. Bacterial scaffold directs pole-specific centromere segregation. Proc Natl Acad Sci U S A. 2014;111:E2046–E2055. doi: 10.1073/pnas.1405188111 24778223 PMC4024888

[77] Bowman GR, Comolli LR, Gaietta GM, Fero M, Hong S-H, Jones Y, et al. Caulobacter PopZ forms a polar subdomain dictating sequential changes in pole composition and function. Mol Microbiol. 2010;76:173–189. doi: 10.1111/j.1365-2958.2010.07088.x 20149103 PMC2935252

[78] Dahlberg PD, Saurabh S, Sartor AM, Wang J, Mitchell PG, Chiu W, et al. Cryogenic single-molecule fluorescence annotations for electron tomography reveal in situ organization of key proteins in Caulobacter. Proc Natl Acad Sci U S A. 2020;117:13937–13944. doi: 10.1073/pnas.2001849117 32513734 PMC7321984

[79] Crymes WB, Zhang D, Ely B. Regulation of podJ expression during the Caulobacter crescentus cell cycle. J Bacteriol. 1999;181:3967–3973. doi: 10.1128/JB.181.13.3967-3973.1999 10383964 PMC93886

[80] Radhakrishnan SK, Thanbichler M, Viollier PH. The dynamic interplay between a cell fate determinant and a lysozyme homolog drives the asymmetric division cycle of Caulobacter crescentus. Genes Dev. 2008;22:212–225. doi: 10.1101/gad.1601808 18198338 PMC2192755

[81] Wheeler RT, Shapiro L. Differential localization of two histidine kinases controlling bacterial cell differentiation. Mol Cell. 1999;4:683–694. doi: 10.1016/s1097-2765(00)80379-2 10619016

[82] Perez AM, Mann TH, Lasker K, Ahrens DG, Eckart MR, Shapiro L. A localized complex of two protein oligomers controls the orientation of cell polarity. MBio. 2017;8: doi: 10.1128/mBio.02238-16 28246363 PMC5347347

[83] Jiang C, Brown PJB, Ducret A, Brun YV. Sequential evolution of bacterial morphology by co-option of a developmental regulator. Nature. 2014;506:489–493. doi: 10.1038/nature12900 24463524 PMC4035126

[84] Wagner JK, Brun YV. Out on a limb: how the Caulobacter stalk can boost the study of bacterial cell shape. Mol Microbiol. 2007;64:28–33. doi: 10.1111/j.1365-2958.2007.05633.x 17376069

[85] Patel A, Malinovska L, Saha S, Wang J, Alberti S, Krishnan Y, et al. ATP as a biological hydrotrope. Science. 2017;356:753–756. doi: 10.1126/science.aaf6846 28522535

[86] Mehringer J, Do T-M, Touraud D, Hohenschutz M, Khoshsima A, Horinek D, et al. Hofmeister versus Neuberg: is ATP really a biological hydrotrope? Cell Rep Phys Sci. 2021;2:100343. doi: 10.1016/j.xcrp.2021.100343

[87] Pattanayak GK, Liao Y, Wallace EWJ, Budnik B, Drummond DA, Rust MJ. Daily Cycles of Reversible Protein Condensation in Cyanobacteria. Cell Rep. 2020;32:108032. doi: 10.1016/j.celrep.2020.108032 32814039 PMC10005845

[88] Viollier PH, Sternheim N, Shapiro L. Identification of a localization factor for the polar positioning of bacterial structural and regulatory proteins. Proc Natl Acad Sci U S A. 2002;99:13831–13836. doi: 10.1073/pnas.182411999 12370432 PMC129783

[89] Hinz AJ, Larson DE, Smith CS, Brun YV. The Caulobacter crescentus polar organelle development protein PodJ is differentially localized and is required for polar targeting of the PleC development regulator. Mol Microbiol. 2003;47:929–941. doi: 10.1046/j.1365-2958.2003.03349.x 12581350

[90] Zhang C, Zhao W, Duvall SW, Kowallis KA, Childers WS. Regulation of the activity of the bacterial histidine kinase PleC by the scaffolding protein PodJ. J Biol Chem. 2022;298:101683. doi: 10.1016/j.jbc.2022.101683 35124010 PMC8980812

[91] Kunz S, Graumann PL. Spatial organization enhances versatility and specificity in cyclic di-GMP signaling. Biol Chem. 2020;401:1323–1334. doi: 10.1515/hsz-2020-0202 32918803

[92] Kreiling V, Thormann KM. Polarity of c-di-GMP synthesis and degradation. microLife. 2023;4:uqad014. doi: 10.1093/femsml/uqad014 37251513 PMC10212136

[93] Glass LN, Swapna G, Chavadi SS, Tufariello JM, Mi K, Drumm JE, et al. Mycobacterium tuberculosis universal stress protein Rv2623 interacts with the putative ATP binding cassette (ABC) transporter Rv1747 to regulate mycobacterial growth. PLoS Pathog. 2017;13:e1006515. doi: 10.1371/journal.ppat.1006515 28753640 PMC5549992

[94] Pu Y, Li Y, Jin X, Tian T, Ma Q, Zhao Z, et al. ATP-dependent dynamic protein aggregation regulates bacterial dormancy depth critical for antibiotic tolerance. Mol Cell. 2019;73:143–156. e4. doi: 10.1016/j.molcel.2018.10.022 30472191

[95] Jin X, Lee J-E, Schaefer C, Luo X, Wollman AJM, Payne-Dwyer AL, et al. Membraneless organelles formed by liquid-liquid phase separation increase bacterial fitness. Sci Adv. 2021;7:eabh2929. doi: 10.1126/sciadv.abh2929 34669478 PMC8528417

[96] Schramm FD, Schroeder K, Alvelid J, Testa I, Jonas K. Growth-driven displacement of protein aggregates along the cell length ensures partitioning to both daughter cells in Caulobacter crescentus. Mol Microbiol. 2019;111:1430–1448. doi: 10.1111/mmi.14228 30779464 PMC6850343

[97] Yoo H, Bard JAM, Pilipenko EV, Drummond DA. Chaperones directly and efficiently disperse stress-triggered biomolecular condensates. Mol Cell. 2022;82:741–755.e11. doi: 10.1016/j.molcel.2022.01.005 35148816 PMC8857057

[98] Zhou Y, Liao H, Pei L, Pu Y. Combatting persister cells: The daunting task in post-antibiotics era. Cell Insight. 2023;2:100104. doi: 10.1016/j.cellin.2023.100104 37304393 PMC10250163

[99] Müller F, Mutch NJ, Schenk WA, Smith SA, Esterl L, Spronk HM, et al. Platelet polyphosphates are proinflammatory and procoagulant mediators *in vivo*. Cell. 2009;139:1143–1156. doi: 10.1016/j.cell.2009.11.001 20005807 PMC2796262

[100] Lempart J, Jakob U. Role of Polyphosphate in Amyloidogenic Processes. Cold Spring Harb Perspect Biol. 2019;11:a034041. doi: 10.1101/cshperspect.a034041 30617049 PMC6496346

[101] Rao NN, Gómez-García MR, Kornberg A. Inorganic polyphosphate: essential for growth and survival. Annu Rev Biochem. 2009;78:605–647. doi: 10.1146/annurev.biochem.77.083007.093039 19344251

[102] Ueber das Nucleïn der Hefe und künstliche Darstellung eines Nucleïns aus Eiweiss und Metaphosphorsäure | CiNii Research. [cited 2024 May 4]. Available from: https://cir.nii.ac.jp/crid/1360011143852358144.

[103] Kern RA, Kingkade MJ, Kern SF, Behrens OK. CHARACTERIZATION OF THE ACTION OF LYSOZYME ON STAPHYLOCOCCUS AUREUS AND ON MICROCOCCUS LYSODEIKTICUS. J Bacteriol. 1951;61:171–178. doi: 10.1128/jb.61.2.171-178.1951 14824095 PMC385981

[104] Friedberg I, Avigad G. Structures Containing Polyphosphate in Micrococcus lysodeikticus. J Bacteriol. 1968;96:544–553. doi: 10.1128/jb.96.2.544-553.1968 5674060 PMC252328

[105] Rao NN, Kornberg A. Inorganic polyphosphate supports resistance and survival of stationary-phase Escherichia coli. J Bacteriol. 1996;178:1394–1400. doi: 10.1128/jb.178.5.1394-1400.1996 8631717 PMC177814

[106] Boutte CC, Henry JT, Crosson S. ppGpp and Polyphosphate Modulate Cell Cycle Progression in Caulobacter crescentus. J Bacteriol. 2012;194:28–35. doi: 10.1128/JB.05932-11 22020649 PMC3256613

[107] Zhang H, Ishige K, Kornberg A. A polyphosphate kinase (PPK2) widely conserved in bacteria. Proc Natl Acad Sci U S A. 2002;99:16678–16683. doi: 10.1073/pnas.262655199 12486232 PMC139203

[108] Zhang H, Gómez-García MR, Shi X, Rao NN, Kornberg A. Polyphosphate kinase 1, a conserved bacterial enzyme, in a eukaryote, Dictyostelium discoideum, with a role in cytokinesis. Proc Natl Acad Sci U S A. 2007;104:16486–16491. doi: 10.1073/pnas.0706847104 17940044 PMC2034253

[109] Thayil SM, Morrison N, Schechter N, Rubin H, Karakousis PC. The role of the novel exopolyphosphatase MT0516 in Mycobacterium tuberculosis drug tolerance and persistence. PLoS ONE. 2011;6:e28076. doi: 10.1371/journal.pone.0028076 22132215 PMC3221697

[110] Choi MY, Wang Y, Wong LLY, Lu B-T, Chen W-Y, Huang J-D, et al. The two PPX-GppA homologues from Mycobacterium tuberculosis have distinct biochemical activities. PLoS ONE. 2012;7:e42561. doi: 10.1371/journal.pone.0042561 22880033 PMC3411833

[111] Alshareedah I, Moosa MM, Raju M, Potoyan DA, Banerjee PR. Phase transition of RNA−protein complexes into ordered hollow condensates. Proc Natl Acad Sci U S A. 2020;117:15650–15658. doi: 10.1073/pnas.1922365117 32571937 PMC7354941

[112] Murata K, Hagiwara S, Kimori Y, Kaneko Y. Ultrastructure of compacted DNA in cyanobacteria by high-voltage cryo-electron tomography. Sci Rep. 2016;6:34934. doi: 10.1038/srep34934 27731339 PMC5059737

[113] Pallerla SR, Knebel S, Polen T, Klauth P, Hollender J, Wendisch VF, et al. Formation of volutin granules in Corynebacterium glutamicum. FEMS Microbiol Lett. 2005;243:133–140. doi: 10.1016/j.femsle.2004.11.047 15668011

[114] Racki LR, Tocheva EI, Dieterle MG, Sullivan MC, Jensen GJ, Newman DK. Polyphosphate granule biogenesis is temporally and functionally tied to cell cycle exit during starvation in Pseudomonas aeruginosa. Proc Natl Acad Sci U S A. 2017;114:E2440–E2449. doi: 10.1073/pnas.1615575114 28265086 PMC5373386

[115] Beaufay F, Amemiya HM, Guan J, Basalla J, Meinen BA, Chen Z, et al. Polyphosphate drives bacterial heterochromatin formation. Sci Adv. 2021;7:eabk0233. doi: 10.1126/sciadv.abk0233 34936433 PMC10954037

[116] van der Lee R, Buljan M, Lang B, Weatheritt RJ, Daughdrill GW, Dunker AK, et al. Classification of intrinsically disordered regions and proteins. Chem Rev. 2014;114:6589–6631. doi: 10.1021/cr400525m 24773235 PMC4095912

[117] Uversky VN. Intrinsically Disordered Proteins and Their “Mysterious” (Meta)Physics. Front Physiol. 2019:7. doi: 10.3389/fphy.2019.0001030800070

[118] Gao C, Ma C, Wang H, Zhong H, Zang J, Zhong R, et al. Intrinsic disorder in protein domains contributes to both organism complexity and clade-specific functions. Sci Rep. 2021;11:2985. doi: 10.1038/s41598-021-82656-9 33542394 PMC7862400

[119] Mao AH, Crick SL, Vitalis A, Chicoine CL, Pappu RV. Net charge per residue modulates conformational ensembles of intrinsically disordered proteins. Proc Natl Acad Sci U S A. 2010;107:8183–8188. doi: 10.1073/pnas.0911107107 20404210 PMC2889596

[120] Das RK, Pappu RV. Conformations of intrinsically disordered proteins are influenced by linear sequence distributions of oppositely charged residues. Proc Natl Acad Sci U S A. 2013;110:13392–13397. doi: 10.1073/pnas.1304749110 23901099 PMC3746876

[121] Bhowmick T, Ghosh S, Dixit K, Ganesan V, Ramagopal UA, Dey D, et al. Targeting Mycobacterium tuberculosis nucleoid-associated protein HU with structure-based inhibitors. Nat Commun. 2014;5:4124. doi: 10.1038/ncomms5124 24916461

[122] Risso-Ballester J, Galloux M, Cao J, Le Goffic R, Hontonnou F, Jobart-Malfait A, et al. A condensate-hardening drug blocks RSV replication *in vivo*. Nature. 2021;595:596–599. 34234347 10.1038/s41586-021-03703-z

[123] Kilgore HR, Mikhael PG, Overholt KJ, Boija A, Hannett NM, Van Dongen C, et al. Distinct chemical environments in biomolecular condensates. Nat Chem Biol. 2024;20:291–301. doi: 10.1038/s41589-023-01432-0 37770698 PMC12181805

[124] Wong F, Zheng EJ, Valeri JA, Donghia NM, Anahtar MN, Omori S, et al. Discovery of a structural class of antibiotics with explainable deep learning. Nature. 2024;626:177–185. doi: 10.1038/s41586-023-06887-8 38123686 PMC10866013

[125] Henry JT, Crosson S. Chromosome replication and segregation govern the biogenesis and inheritance of inorganic polyphosphate granules. Mol Biol Cell. 2013;24:3177–3186. doi: 10.1091/mbc.E13-04-0182 23985321 PMC3806658

