# Supplementary material for: Biomolecular condensates as stress sensors and modulators of bacterial signaling
Source: PLoS Pathog. 2024 Aug 15;20(8):e1012413. doi: 10.1371/journal.ppat.1012413 (PMC11326607; doi:10.1371/journal.ppat.1012413)
Supplement: S1 Fig — The numbers above each bar represent the total number of IDPs that were included in the analyses. Each IDP’s GO term annotation was obtained from Uniprot. GO term categories that occupied less than 2% in all species are excluded from the graph. (PDF) [file ppat.1012413.s001.pdf]

Biomolecular condensates as stress sensors and modulators of bacterial signaling

Moeka Sasazawa[1], Dylan T. Tomares[2,+], W. Seth Childers[2,*], Saumya Saurabh[1,*]

Supplementary information

S1 Figure

Figure S1. Bar plots showing Gene Ontology (GO) term analyses of IDPs from ESKAPE pathogens. The numbers above each bar represent the total number of IDPs that were included in the analyses. Each IDP's GO term annotation was obtained from Uniprot. GO term categories that occupied less than 2% in all species are excluded from the graph.

9

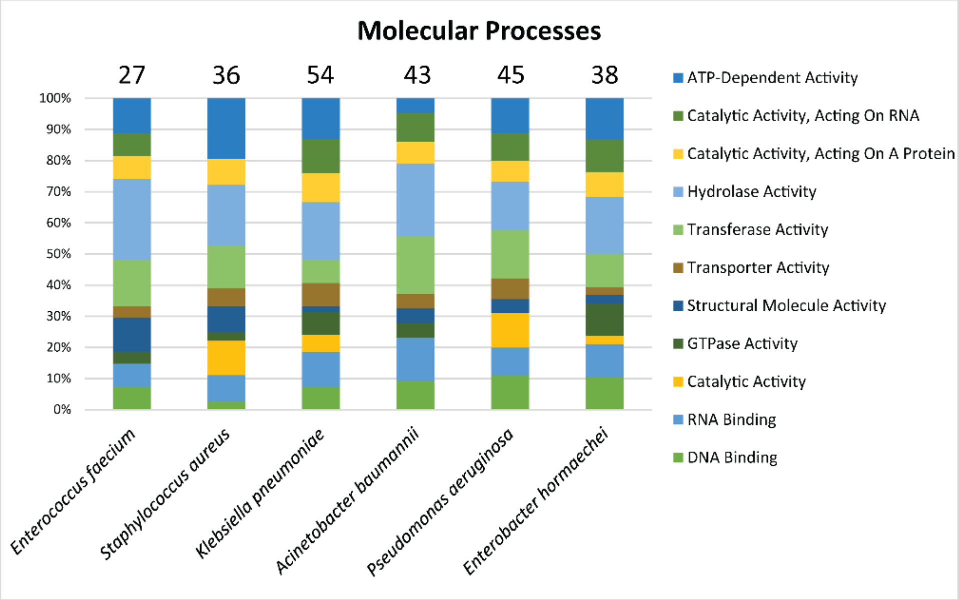

Molecular Processes
27
36
54
43
45
38
100%
90%
80%
70%
60%
50%
40%
30%
20%
10%
0%
Enterococcus faecium
Staphylococcus aureus
Klebsiella pneumoniae
Acinetobacter baumannii
Pseudomonas aeruginosa
Enterobacter hormaechei
ATP-Dependent Activity
Catalytic Activity, Acting On RNA
Catalytic Activity, Acting On A Protein
Hydrolase Activity
Transferase Activity
Transporter Activity
Structural Molecule Activity
GTPase Activity
Catalytic Activity
RNA Binding
DNA Binding

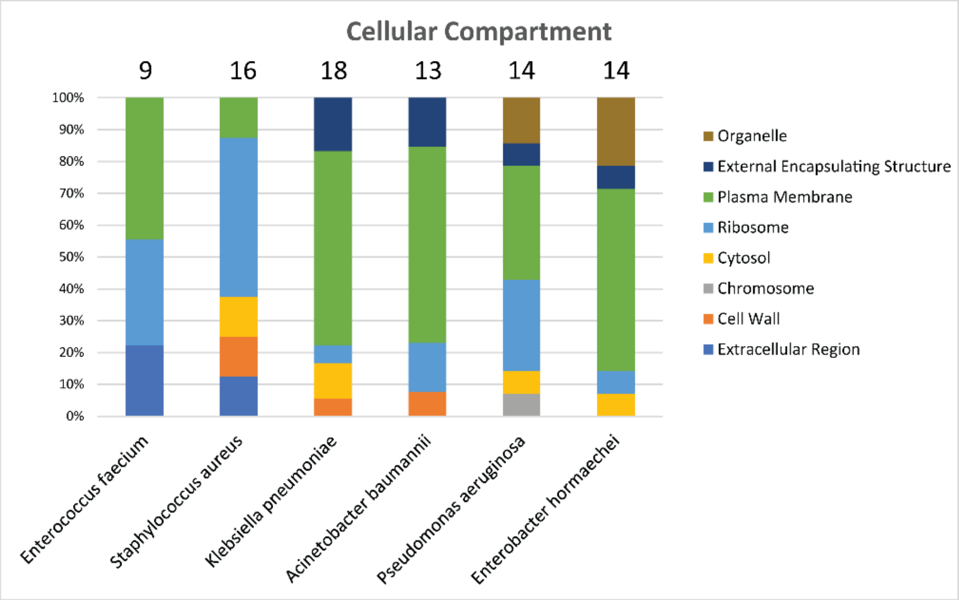

Cellular Compartment
9
16
18
13
14
14
100%
90%
80%
70%
60%
50%
40%
30%
20%
10%
0%
Enterococcus faecium
Staphylococcus aureus
Klebsiella pneumoniae
Acinetobacter baumannii
Pseudomonas aeruginosa
Enterobacter hormaechei
Organelle
External Encapsulating Structure
Plasma Membrane
Ribosome
Cytosol
Chromosome
Cell Wall
Extracellular Region

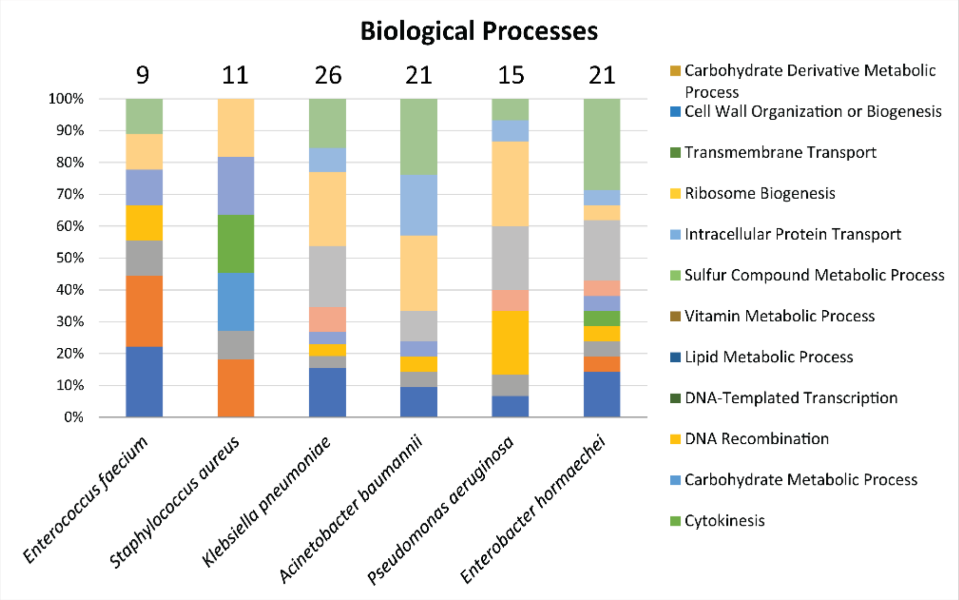

Biological Processes
9
11
26
21
15
21
100%
90%
80%
70%
60%
50%
40%
30%
20%
10%
0%
Enterococcus faecium
Staphylococcus aureus
Klebsiella pneumoniae
Acinetobacter baumannii
Pseudomonas aeruginosa
Enterobacter hormaechei
Carbohydrate Derivative Metabolic Process
Cell Wall Organization or Biogenesis
Transmembrane Transport
Ribosome Biogenesis
Intracellular Protein Transport
Sulfur Compound Metabolic Process
Vitamin Metabolic Process
Lipid Metabolic Process
DNA-Templated Transcription
DNA Recombination
Carbohydrate Metabolic Process
Cytokinesis
