# Supplementary material for: Biomolecular condensates as stress sensors and modulators of bacterial signaling
Source: PLoS Pathog. 2024 Aug 15;20(8):e1012413. doi: 10.1371/journal.ppat.1012413 (PMC11326607; doi:10.1371/journal.ppat.1012413)
Supplement: S1 Table — (PDF) [file ppat.1012413.s002.pdf]

Biomolecular condensates as stress sensors and modulators of bacterial signaling

Moeka Sasazawa[1], Dylan T. Tomares[2,+], W. Seth Childers[2,*], Saumya Saurabh[1,*]

Supplementary information

Table S1: Abundance of IDPs in pathogens, free-living and endosymbiotic microbes

Bacteria	Total Proteins	IDPs with >50 disordered residues	IDPs with >30 disordered residues
Enterococcus faecium	2825	74	153
Staphylococcus aureus	2622	90	175
Klebsiella pneumoniae	5137	80	186
Acinetobacter baumannii	3785	80	178
Pseudomonas aeruginosa	5863	144	318
Enterobacter spp.	4070	93	195
Caulobacter crescentus	3859	143	330
Saccharomyces cerevisiae (Baker's yeast)	6245	1442	2079
Wolbachia endosymbiont of Cylisticus convexus	1643	31	69
Rickettsia rickettsii	1343	18	38
Rickettsia felis	1619	29	57
Rickettsia conorii	1371	18	37
Rickettsia argasii	1689	21	51
Ehrlichia chaffeensis	1100	24	53
Anaplasma phagocytophilum	1614	49	95
